# Value-based decision-making between affective and non-affective memories

**DOI:** 10.1016/j.isci.2024.109329

**Published:** 2024-02-24

**Authors:** Erdem Pulcu, Calum Guinea, Hannah Clemens, Catherine J. Harmer, Susannah E. Murphy

**Affiliations:** 1 University of Oxford, Department of Psychiatry, Warneford Hospital, OX3 7JX Oxford, UK

**Keywords:** Neuroscience, Social sciences, Psychology

## Abstract

Affective biases can change how past events are recalled from memory. To capture mechanisms underlying affective memory formation, recall, and bias, we studied value-based decision-making (VBDM) between reward memories encoded in different mood states. Our findings suggest that following discrete affective events, created by large magnitude wins and losses on a Wheel of Fortune (WoF), healthy volunteers display an overall positive memory bias [favoring higher probability shapes learned after a WoF win compared with those learnt after a WoF loss outcome]. During this VBDM process, participants’ pupils constrict before decision-onset for higher-value choices, and remained dilated for a sustained period after choice. Sustained pupil dilation was particularly sensitive to the reward values of abstract memories encoded in a positive mood. Taken together, we demonstrate that experimentally induced affective memories are recalled with a positive bias, and pupil-linked central arousal systems are actively engaged during VBDM between affective and non-affective memories.

## Highlights

• Discrete affective events do not influence reinforcement-learning performance
• Humans show nonlinear preferences between experimentally induced affective memories
• Memory-guided decision-making engages central arousal systems and pupil dilation
• Pupil dilation is sensitive to reward values of memories encoded in a positive mood

## Graphical abstract

**Figure:**
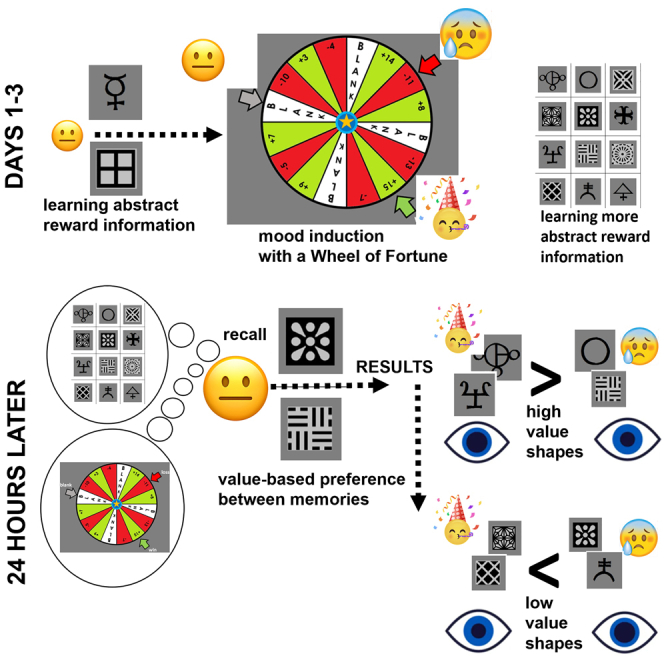

## Introduction

Human life is arguably the most complex in the animal kingdom, enriched by our ability to express and infer from others a wide spectrum of emotions. The breadth of this affective repertoire, along with our tendency to process positive and negative information asymmetrically,^1^^,^^2^ introduces biases that can shape not only our present experiences, but also how we recall events from the past. Asymmetries in affective information processing are commonly referred to as “affective biases.” Existing literature has generally observed a positive affective bias in nonclinical populations, resulting in a preference for positive events across multiple domains including perception, attention, reinforcement learning (RL), and memory.^3^^,^^4^ Conversely, in psychiatric conditions such as major depressive disorder (MDD), negative affective biases (i.e., preferential processing of negative relative to positive information)^5^^,^^6^^,^^7^^,^^8^^,^^9^ have been shown to play a role in the development and maintenance of symptoms.^10^^,^^11^^,^^12^ The effect of these affective biases and how incidental changes in effect influence immediate decision-making^13^ are reasonably well characterized, yet the computational and physiological mechanisms underlying how discrete affective events (e.g., unexpected positive or negative events) induce biases that can influence learning and subsequent memory recall are less well understood.

Previous literature suggests that the recall of information from memory leads to pupil dilation.^14^^,^^15^ Existing literature suggests that the pupil dilation is under the influence of a number of neurotransmitter systems such as norepinephrine, serotonin, and acetylcholine.^16^ However, recent studies investigating the physiological correlates of autobiographical memory recall did not reveal differences in relation to the affective valence of events during retrieval.^17^

**Figure 1:**
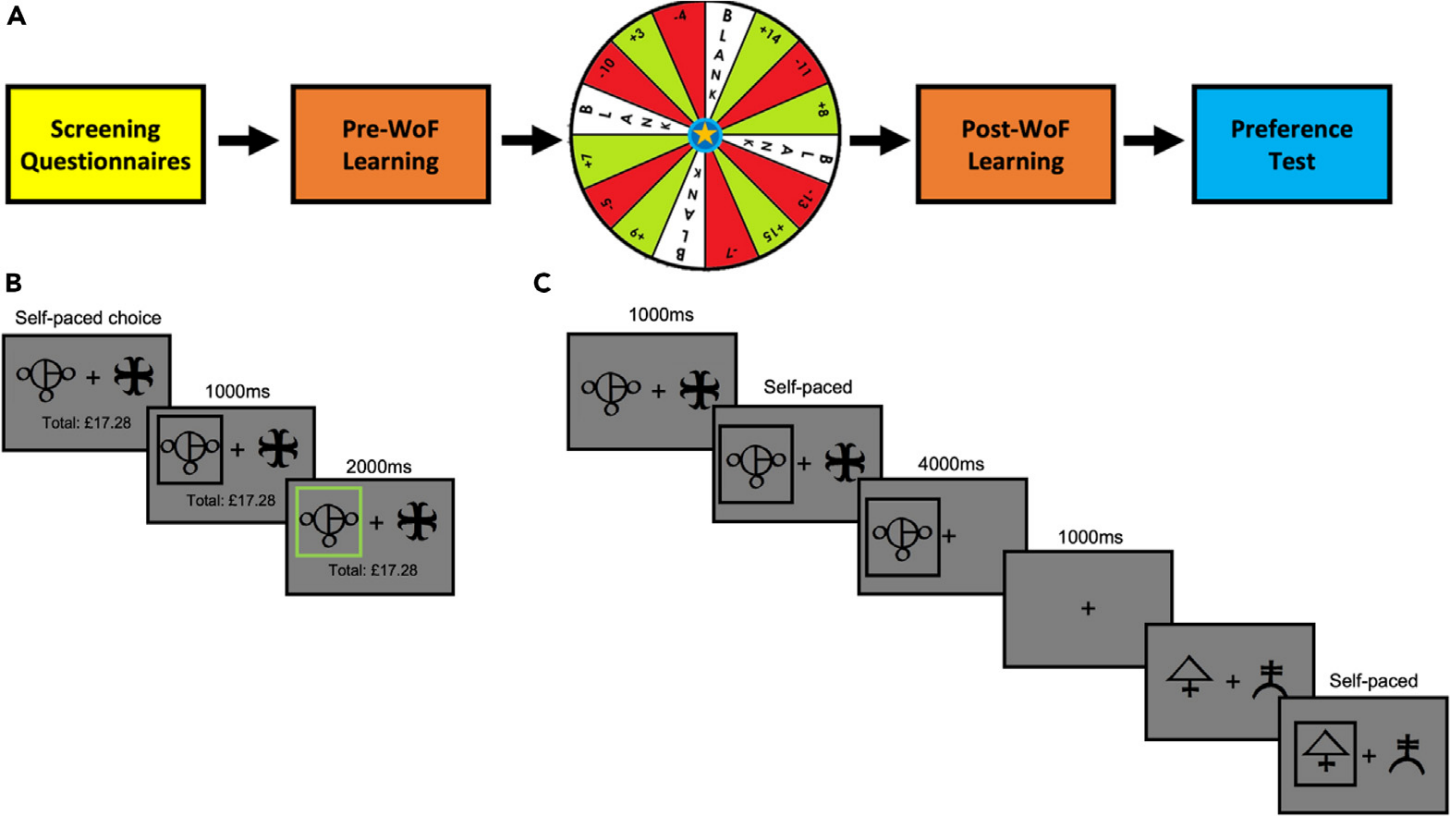
General outline of the experiments (A) Demonstrates the shared structure of Study 1 and 2. Participants were first screened for inclusion/exclusion criteria and then completed a set of questionnaires (see Table 1). They subsequently learned the stimulus-reward associations of a set of stimuli prior to the WoF draw before experiencing a single WoF outcome, and concluded by learning the stimulus-reward associations of another set of stimuli. Their participation in the experiment concluded with a preference test where previously learned stimulus-reward associations were tested by repeatedly pairing all possible combinations of the stimuli together and asking participants to choose which one they thought was most rewarding. (B) A schematic of a single learning trial in Study 1 and 2. Participants were presented with a pair of stimuli and instructed to choose between them based on which one they thought would be rewarded. This choice was self-paced and once a choice was made, this was indicated on the screen by overlaying a black box around the chosen stimulus. If this stimulus was rewarded, this box would turn green. If the choice was incorrect, a green frame appeared around the unchosen shape. On each trial, one of the shapes was linked to a “win” outcome (+2 pence) and the other shape resulted in no monetary gain. The win and null outcomes were dependent on each other (probabilities add up to 1). Using trial and error, participants could learn the reward probability associated with each shape. This information could then be used to maximize their monetary reward. Participants started with £15 and their running total, displayed below the fixation cross for the duration of every trial, updated by 2p for each correct choice made. Incorrect choices did not have any monetary effect on participants’ running total. Within each RL block the shape pair always stayed the same, such that participants learned about only 2 shapes per block, with their probabilities always summing up to 1 (e.g., 0.9 vs. 0.1). (C) A schematic of the preference test. Here, participants are shown all possible pairs of the stimuli they encountered during learning and instructed to choose between them based on which one they thought was most rewarded during learning. On each trial, participants are shown a pair of stimuli and make a self-paced choice after the stimuli are on-screen for 1000 ms. Once a decision is made, the unchosen shape would disappear from the screen. Here, participants did not see their running total to avoid secondary learning effects through feedback. Examples from 2 trials are given for illustrative purposes. Four examples of actual fractals used in the studies are shown in panels B and C.

In this work, we sought to develop an experimental analogue of episodic affective (i.e., negative and positive) events by manipulating the magnitude of unexpected financial outcomes participants experience in a Wheel of Fortune (WoF) draw. WoF draws occurred between reinforcement-learning (RL) blocks in which participants were asked to learn the probability of rewards associated with abstract fractals through trial-and-error (Figure 1). Shape pairs were presented one at a time in individual RL blocks. At least 24 h after the last training day [in which the participants completed the individual RL blocks], participants were asked to choose between random pairings of all the fractals they learnt across all training days. Here, it was important for participants to recall reward contingencies associated with abstract fractals, as their final cumulative reimbursement depended on how well they identified the shapes with higher reward probability. Therefore, we refer to this memory-based decision process as “value-based recall.” However, we expected that the outcome of the WoF draws experienced during the training days and in between individual RL blocks, would affect participants’ memory of the learnt fractals, and therefore subsequent judgements. For example, shapes learnt after a win outcome in the WoF might be remembered as more positive than they actually were, or alternatively shapes following a loss outcome in the WoF might be remembered as more negative, due to the feedforward mood effects of unexpected wins and losses in the WoF. Further details of the experiment in addition to this general outline are given in the Figure 1 legend later in discussion and in STAR methods.

Across two studies, we present evidence suggesting that for experimentally induced affective memories, human behavior is described by nonlinear value-based decision-making models and pupil dilation is sensitive to the reward values of abstract affective information encoded under positive mood even 24 h later.

## Results

### Participants and demographics

45 healthy volunteers took part in Study 1 and 74 healthy volunteers took part in Study 2. A summary of participant demographics and baseline self-report questionnaire data is presented in Table 1.

**Table 1:** Participant Demographics and questionnaire scores in the two studies

Measure	Study 1 (n = 45)	Study 2 (n = 74)
	Mean ± SD	Mean ± SD
Age	29.33 ± 7.98	33.94 ± 7.95
Gender, female	31 (69%)	62 (84%)
Years of education	16.53 ± 3.00	N/C
Trait-STAI	32.51 ± 9.11	32.76 ± 10.94
State-STAI	29.31 ± 7.39	29.83 ± 11.1
BDI	3.56 ± 4.00	5.15 ± 5.46
BAS Drive	6.64 ± 2.31	8.2 ± 2.75
BAS Fun	7.16 ± 2.01	8.45 ± 2.71
BAS Reward	12.02 ± 1.71	13.05 ± 2.59
BIS	15.69 ± 3.41	16.15 ± 3.92
MDQ	3.02 ± 3.65	4.08 ± 3.68
PANAS positive affect	34.64 ± 6.97	31.54 ± 10.4
PANAS negative affect	17.67 ± 12.40	12.21 ± 4.51

Trait STAI, Spielberger State-Trait Anxiety Inventory, trait form; State STAI, Spielberger State-Trait Anxiety Inventory, state form; BDI, Beck Depression Inventory; BAS, Behavioral Activation; BIS, Behavioral Inhibition; MDQ, Mood Disorder Questionnaire; PANAS, Positive and Negative Affect Schedule.

N/C, not collected.

To assess the effect of mood on memory bias the first stage involved learning/encoding [of abstract information] following an experimental mood manipulation. The Wheel of Fortune manipulation used here to induce positive and negative changes in mood, yielded similar results to previous work.^18^^,^^19^ In order to focus on memory-guided decision-making and its corresponding physiology, we report a brief summary of the mood effects and learning behavior later in discussion, and further details are communicated in the supplementary results section.

### Wheel of Fortune outcomes influence mood but not learning performance

Participants’ ratings indicated that they felt significantly happier immediately after winning in the WoF and felt significantly less happy immediately after losing (see supplementary results). The WoF was therefore effective at modulating mood in the expected direction, and mood effects persisted until the end of the last learning block (Figure S1).

**Figure 2:**
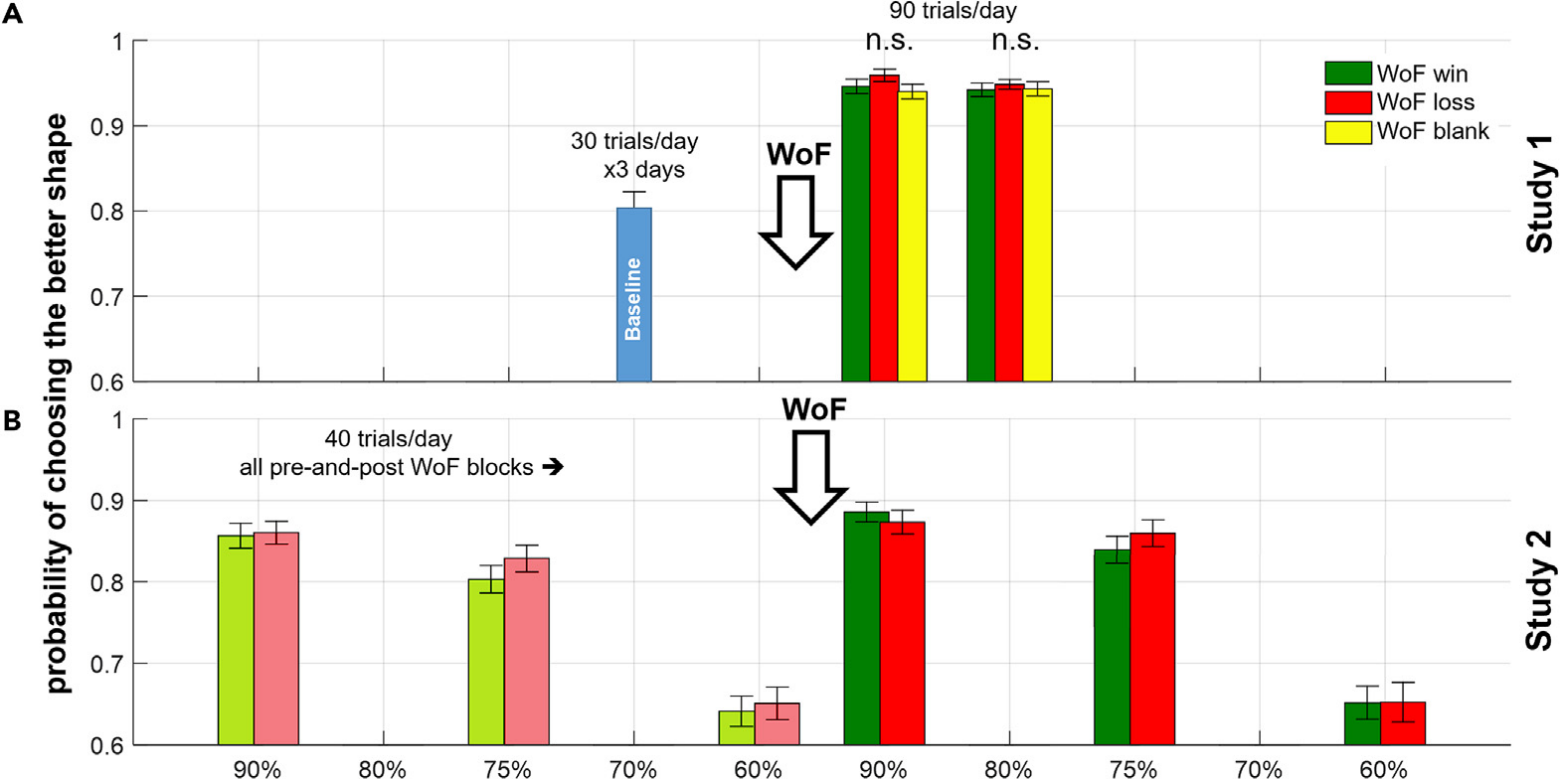
Summaries of probability of choosing the higher probability shape (A) In Study 1, there were no significant differences in participant learning behavior after the WoF, irrespective of the valence of WoF outcome. The single blue bar at 70% before the WoF shows the baseline condition. (B) In Study 2, we were able to compare pre-and-post-WoF learning behavior as the reward probabilities were tightly controlled. A repeated measures ANOVA model indicated a significant main effect of WoF influencing participant choice behavior, reflecting an increased probability of choosing the shape associated with a high probability of reward post-WoF, irrespective of valence of the WoF outcome. Downward arrow labeled with WoF indicates the point in which participants experienced the WoF draw within the course of their daily learning sessions. Note that, Study 1 had 3 training days, whereas the Study 2 only had 2 training days, therefore only win and loss outcomes in the WoF (i.e., blank WoF outcome was omitted in Study 2). In both panels, error bars reflect ±1 SEM. Probabilities on the x axis reflect reward probabilities from Table 2. Bar face colors: blue (baseline condition, only Study 1), yellow (neutral, only Study 1), green (win, both studies), red (loss, both studies). Empty spaces on the x axis intend to highlight different reward probability levels covered in each study. X axis labels also highlight the order of RL blocks [with respect to their reward probabilities] participants completed within each training day. In the bottom panel, the lighter colored bars highlight the pre-WoF blocks.

Across both studies, participants were able to correctly identify the high probability shape in each RL block. In Study 1 and Study 2 there was no significant effect of the WoF outcome (win or loss) on participants’ tendency to choose the higher probability shapes during reinforcement learning. (Study 1: F(2,78) = 2.045, p = 0.136, Figure 2A; Study 2: F(1,65) = 2.439, p = 0.123, Figure 2B). These results suggest that preference differences that we report in the subsequent sections occurring 24- and 48-h post-learning do not originate from changes in learning behavior in the task blocks presented immediately after the WoF draw. Further results from exploratory model-free and model-based analyses of learning behavior are also reported in supplementary results and Figures S2–S6.

### An overarching influence of reward probability on value-based recall

In the preference tests 24 h and 48 h after learning, participants were asked to choose between pairs of shapes that they had previously seen to indicate which shape they thought was associated with a higher probability of reward during learning/encoding. As mentioned in the introduction section, participants final reimbursement depended on their performance in selecting the fractals they thought were associated with higher reward probabilities. As expected, there was a main effect of reward probability on shape preference in both Study 1 and 2 (F(1,39) = 48.100 and 20.571, respectively, both p <0 .001), with participants more likely to choose shapes that had been associated with a high probability of reward during encoding. In Study 1, there was also a significant interaction between probability and WoF outcome valence (F(2,78) = 11.316, p <0 .001, Figure S7A), which reflected valence-specific effects for low/high probability shapes. This was seen in both preference tests (24 h and 48 h post-learning). To further explore this interaction between WoF outcome valence and reward probability, we fit a multilevel [logistic] regression model. In this model the positive effect of WoF outcome on reward probability was coded as +1 for high probability shapes (>0.50) and as −1 for low probability shapes (<0.50). The choices were then coded as 1s and 0s. We fitted this model separately for all satisfying trials and all participants choosing directly between (i) WoF-win versus WoF-neutral, (ii) WoF-loss versus WoF-neutral, and finally (iii) WoF-win versus WoF-loss outcome shapes. These analyses indicated significant effects of WoF outcome on high reward probability shapes in WoF-win versus WoF-neutral (t(42) = 4.578, p <0 .001), WoF-loss versus WoF-neutral (t(42) = 2.048, p = 0.047), and WoF-win versus WoF-loss outcome shapes (t(42) = 3.235, p = 0.002).

WoF outcome valence by reward probability interaction was not seen in Study 2. This may be due to design differences in between Studies 1 and 2. Study 1 had an asymmetrical design in terms of reward probabilities for baseline/pre-WoF blocks (see Table 2 for details).

**Table 2:** Differences between the designs of Study 1 and 2

Design feature	Study 1	Study 2
Overall experiment duration (days)	5	3
Number of learning days	3	2
Number of preference test days	2	1
Number of pre-WoF learning blocks	1	3
Number of pre-WoF trials/repetitions per block	30	40
Pre-WoF reward probabilities (in the order of appearance)	[0.7 & 0.3]	[0.9 & 0.1], [0.75 & 0.25], [0.6 & 0.4]
Number of post-WoF learning blocks	2	3
Number of post-WoF trials/repetitions per block	90	40
Post-WoF reward probabilities (in the order of appearance)	[0.9 & 0.1], [0.8 & 0.2]	[0.9 & 0.1], [0.75 & 0.25], [0.6 & 0.4]
Mood sampling frequency	Start, pre-WoF, post-WoF, End	Before and after every block
Total number of mood samples per day	4	8
Possible WoF outcomes	Win, loss, blank	Win or loss
Eye tracking during preference test	Yes (Day 1)	No

Table 2 lists the differences between Study 1 and 2. As in Figure 1, the overall experimental procedure of the two studies was identical, however there were a number of differences between the two studies, including but not limited to: the overall duration, the reward probabilities of learned stimuli pre and post-WoF and the number of trials per learning block. Note that pre-WoF/baseline block of 70/30% contingency was repeated identically on each of 3 training days in Study 1, such that the total repetitions between pre-and-WoF RL shapes would be identical by the end of the training days (e.g., 30x3 baseline 70% versus 90x1 WoF-win 90%).

### Humans display non-linear preferences between affective memories during value-based recall

In simple rodent assays investigating the role of affective memories,^20^ or previous human studies which covered only two points along the reward probability spectrum,^18^^,^^19^ WoF effects have been analyzed by relying on equal value comparisons between pre-and-post WoF shapes (e.g., 70% post-WoF win shapes vs. 70% pre-WoF shapes). These results are reported in Table S1 and in Figure S8. However, our studies had much wider coverage of the probability spectrum, achieved by using 14 shapes in Study 1, and 24 shapes in Study 2. This means that in Study 1 there could be [14 × 13 = ]182 individual comparisons, and equal value comparisons would only account for 12 of these “contrasts.”

**Figure 3:**
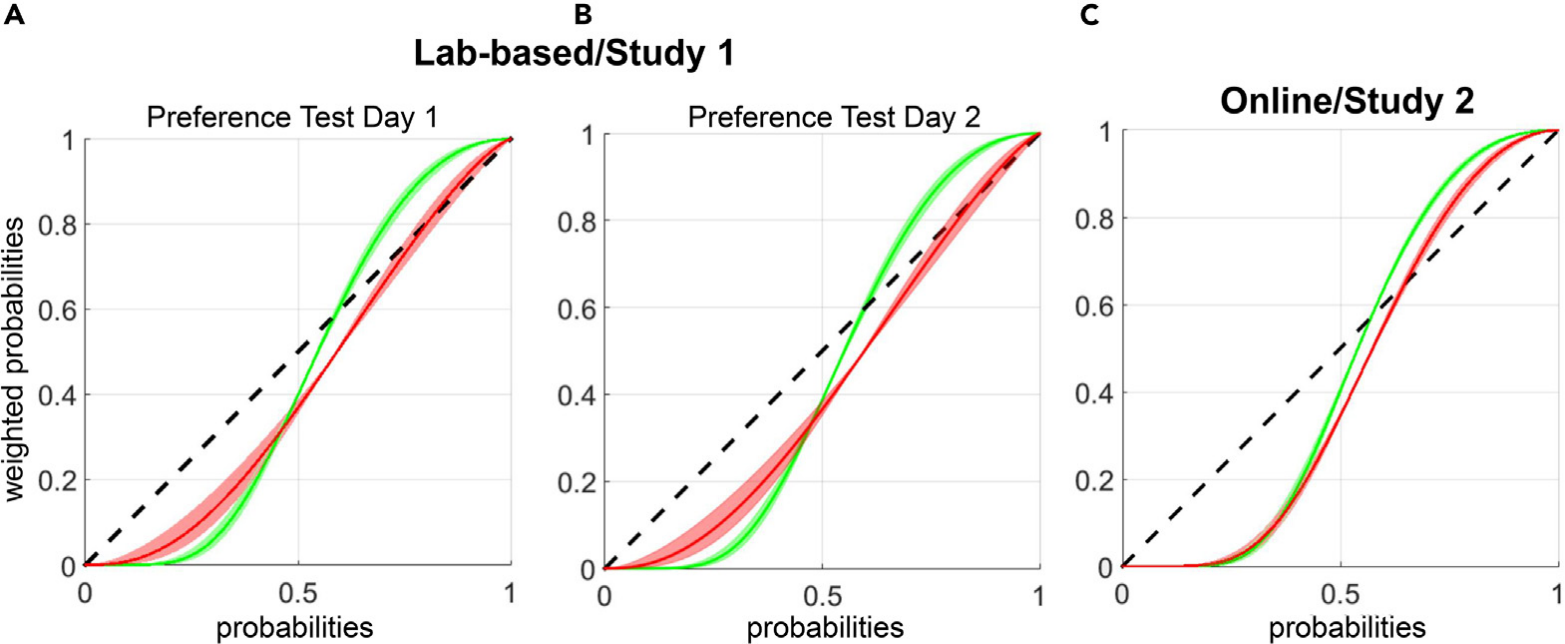
Positive bias in memory-guided value-based decision-making (A and B) Consistent with the model-free results reported in Table S1 and Figure S6, model-based results show consistent effects between preference test days 1 and 2 (e.g., the direction of t-test comparisons of equal-probability shapes reported on rows 1–4 of Table S1 are in full agreement with the win and loss subjective probability trajectories revealed by the model). (C) Model-estimated subjective reward probabilities during value-based recall between affective memories in the online study (Study 2) in which all stimuli were presented randomly on each side of the screen. The difference between win and loss trajectories become more evident for higher probability shapes, which were sampled more frequently during the encoding stage. Shading around the population mean denotes ±1 SEM. Across all panels green lines designate the probability weighting associated with the WoF win, whereas red lines designate the probability weighting associated with the WoF loss outcome.

Rather than solely relying on equal value comparisons, we sought to construct a model of human value-based recall of affective memories, by analysing participant choice behavior in the preference tests with computational modeling (see STAR methods and the supplemental information for details). Here, we propose a simple value-based decision-making model in which participants choose between shape pairs based on the difference between their reward probabilities such that shapes with higher reward probabilities should be preferred over those with lower reward probabilities. In order to account for subjectivity/distortions in how each participant recalled reward probabilities associated with learned shapes we used a non-linear (i.e., an exponential-logarithmic) 2-parameter probability weighting function (Figure 3). This model is described in supplemental information (see Equations 6, 7, and 8).

First, we report the goodness-of-fit metrics for our proposed probability weighting model. Considering that in the majority of the preference test trials, the expected absolute value difference between the options were zero (e.g., 60% post-WoF win vs. 60% post-WoF loss shapes, Figure S9), this would normally warrant random (i.e., 50-50) choices between these options. Consequently, a random decision-making model would have a theoretical benchmark log likelihood value of −0.69 (i.e., log(0.50)). This model would be mathematically equivalent to a model without any probability weighting parameters and no inverse temperature term (i.e., β = 1). We tested how well our stochastic choice model for the preference test performs against this benchmark, which would also define an index of model goodness-of-fit. Across both Study 1 and Study 2 this stochastic choice model performed significantly better than this benchmark (all t > 8.2, all p <0 .001). This means that the model can capture the subjective valuations underlying binary decision-making between affective and non-affective memories with, for the majority of the trials, no objective value difference. This comparison also indicates that participants were attentive during the preference test and did not make decisions randomly, rather their decisions followed the proposed expected value function that relies on weighted probabilities. We further tested our proposed model against a model with no probability weighting parameters but only a single inverse temperature term to capture inherent choice stochasticity in binary decision-making. In both studies, our proposed model with weighting parameters fitted the data significantly better than a model without any weighting parameters and only an inverse temperature term (all t > 5.03, all p <0 .001). For completeness, we performed parameter recovery simulations^21^ which demonstrated that parameter recovery adequately captures the essence of the original findings (e.g., crossover between post-WoF win and post-WoF loss related probability weighting trajectories from low to high probability range) and recapitulates the behavioral results (Figure S10). First, 2^nd^, and 3^rd^ quartile values of model parameters are reported in Table S2.

The area under the curve (AuC) between probability weighting curves [that track how the probabilities associated with shapes following a win or a loss WoF outcome are utilised non-linearly during binary decision-making] indicated an overall positive bias in value-based recall; Study 1 t(42) = 1.77, p = 0.08 (day 1) and t(42) = 2.57, p = 0.014 (day 2); Study 2: t(68) = 2.027, p = 0.047, Figure 3). These results further demonstrate that discrete positive events (i.e., winning on a WoF draw relative to losing) influence subsequent value-based recall. This adds and complements the findings related to the main effect of reward probability on participant choice behavior reported in the preceding section (i.e., shapes associated with higher reward probabilities which were sampled more frequently during the encoding stage, were preferred more strongly in the preference stage). Modeling choice behavior revealed that the affective influence associated with the WoF win outcome acted in a manner that further augments the subjective reward probabilities of these options during value-based recall (Figure 3). Furthermore, this model-based AuC measure of positive bias correlated highly significantly with its model-free equivalent (e.g., the difference in the number of times high probability win shapes were chosen minus the number of times high probability loss shapes were chosen): r(44) = 0.822 and 0.844 in Study 1 with 14 abstract shapes (day 1 and day 2, respectively) and r(73) = 0.652 in Study 2 with 24 abstract shapes (all p <0 .001).

One definition of affective bias is how affective information bleeds over and taints otherwise neutral information [subconsciously].The trajectories of the probability weighting function predict that high probability baseline shape (i.e., 70%) would be preferred more strongly when they were presented on the side of the post-WoF win outcome shapes such that the reward probability associated with the baseline shape will be augmented, whereas high probability baseline shapes would be under-weighted when they are presented on the side associated with post-WoF loss shapes (e.g., red curve and SEM shading in Figure 3A). The model-free analogue of this process confirmed that high probability post-WoF neutral shapes were preferred significantly more frequently when they were presented on the side of the post-WoF win shapes (F(1,44) = 8.531, p = 0.005). Taken together, these findings indicate a close agreement between model-based and model-free metrics of affective bias.

### Null effect of depressive symptoms on positive memory bias in healthy volunteers

Although we did not recruit participants specifically to include high versus low depressive symptom severity in this cohort, we explored the degree to which self-reported symptoms of depression (23% had a 0 score on BDI) relate to our index of positive memory bias (i.e., AuC measure described above). We combined the data from both cohorts due to qualitatively overlapping results and fitted a multiple linear regression model using BDI scores as a normalized regressor, while controlling for the effects of age, gender, and WoF order. Intriguingly, the only significant regressor from this model is the intercept (t(112) = 2.458, p = 0.016), whereas self-reported depression scores did not seem to influence this metric (t(112) = -0.246, p = 0.81). A significant intercept in this model suggests that the AuC measure described in the preceding section is greater than zero, and confirms positive memory bias.

### Value-based decision-making between affective memories engages the pupil-linked central arousal systems prior to choice

**Figure 4:**
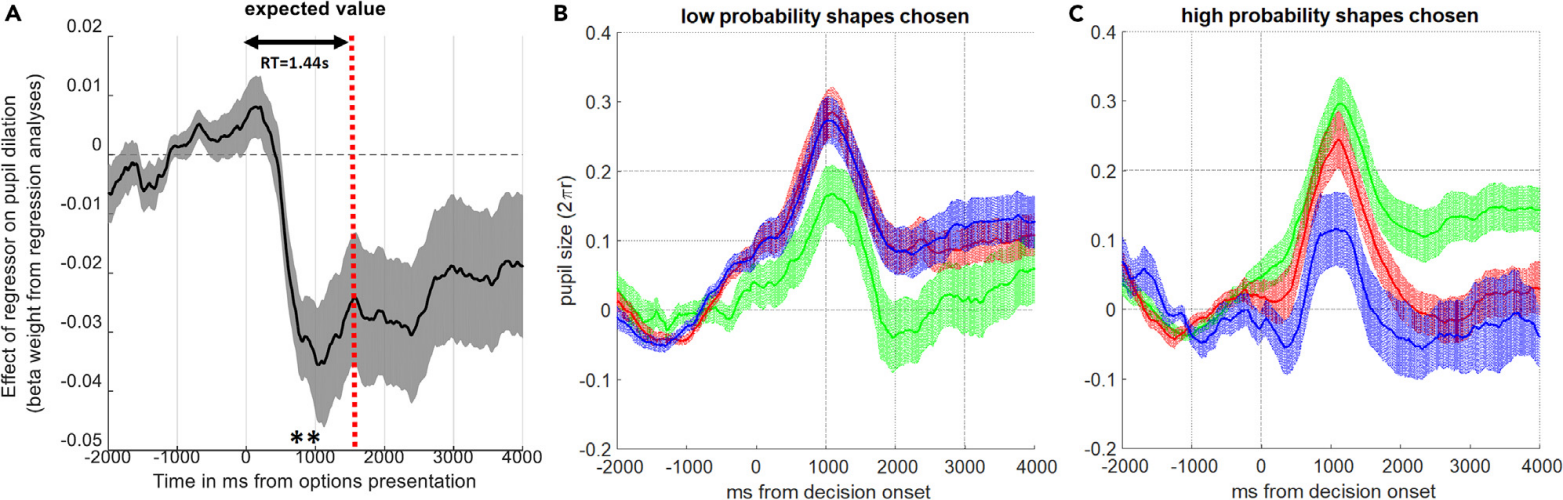
Pupil dilation before and after choice (A) A multiple linear regression analysis of the pupillary response suggests that pupil dilation negatively correlates with the expected values of chosen shapes based on the model shown in Figure 3. The vertical red dashed line marks the average response time (RT = 1.44 s) during value-based decision-making. The black line with gray error shading shows the time evolution of the regression coefficients. ∗∗p <0 .01. (B) After the decision onset and during the outcome delivery period (i.e., once a decision has been made and a low probability option is selected [irrespective of the probability associated with the unchosen option]) low probability post-WoF win shapes lead to a smaller pupil dilation (green line with associated shading) relative to post-WoF loss (red line) and post-WoF neutral (blue line) shapes (the difference between green lines versus the red/blue line). (C) For high probability chosen shapes [irrespective of the probability associated with the unchosen shape, whether it is high or low on any given trial], this physiological signature is reversed, such that win shapes lead to larger pupil dilation. Note that as soon as the decision was made (0 ms), the unchosen option was removed from the screen, and the chosen option was displayed for further 4 s. No feedback was given on the accuracy of the choice to avoid additional learning during the preference test. In all panels, shading around the population mean denotes ±1 SEM.

During the first preference test of Study 1, pupillometry data were collected across the entire decision process. First, we tested the prediction that pupil dilation encoding the expected value of to-be-chosen options should peak before the choice onset. In this analysis we first controlled for the expected value difference between the options as a proxy for choice difficulty. Secondly, to minimize the possibility that luminosity associated with the appearance of fractals that we used (always drawn in black against the same light gray background) may be the main driver of these results, we computed the number of black pixels in each fractal in MATLAB and used these values in a trial wise fashion as additional regressors in the pupillometry model. Prior to choice, after controlling for the expected value difference between presented options as a proxy for choice difficulty and differences in overall luminosity between the shapes, the expected value of the to-be-chosen options estimated by the computational model reported above was significantly negatively correlated with pupil dilation (t(38) = −3.23, p =0 .003, Figure 4A, peak pupil dilation between 560 and 1000 ms, significance at peak p = 3x10^−5^). This suggests that choosing shapes associated with lower expected value computed based on the decision model (Figure 3A) is associated with pupil dilation and provides a physiological correlate of this model as the decision unfolds. We further investigated the amount of time participants spent gazing/evaluating the shapes as a proportion of the total reaction time. We did this analysis separately for each shape reward probability, and repeated the same analysis using absolute value differences as a general index of choice difficulty (Figure S11). These analyses indicated a main effect of reward probability (F(1,5) = 3.013, p = 0.023) only when the data is sliced with respect to reward the probability of shapes (irrespective of choice and the unchosen shape probability, Figure S11A), otherwise there was no main effect of WoF-outcome valence or any valence by probability interactions (all F < 1.853, all p >0 .129).

### Sustained arousal for affective memories reflects the behavioral index of positive memory bias

**Figure 5:**
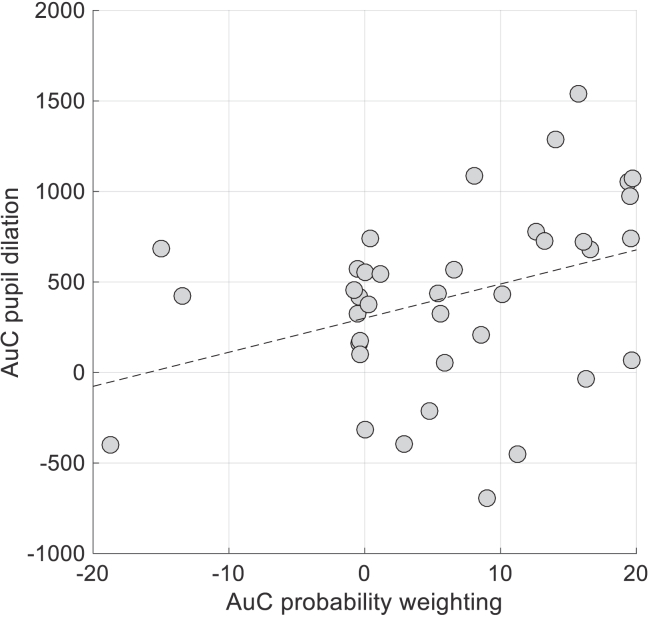
Sustained pupil dilation is related to the index of positive memory bias Area under the curve (AuC) computed from probability weighting curves (Figure 3A, Study 1 assessment with pupillometry) correlates with the AuC accounting for a sustained pupil dilation during the post-decision epoch for post-WoF win related shapes, relative to post-WoF loss and post-WoF neutral shapes (Figures 4B and 4C). Dashed black line designates the least-squares regression line (r(36) = 0.362, p = 0.025). The correlation remains significant in an exploratory analysis excluding three potential outliers with a negative behavioral AuC index (r(33) = 0.391, p = 0.02).

Next, we visually assessed pupil time courses, which were recorded while participants continued observing the chosen shapes. We observed that after a choice had been made, affective memories had different physiological properties. Two 3×4 rmANOVA (3 levels of valence (i.e., win, loss, null WoF outcomes) × 4 1-s timebins in the outcome delivery period) models indicated a significant main effect of WoF outcome valence (F(2,74) = 5.198, p = 0.008 for low probability and F(2,74) = 6.782, p = 0.002 for high probability shapes Figures 4B and 4C, note that similarly significant results can be obtained even if baseline shapes are also included in the model, which would then be a 4×4 rmANOVA model). Here, note that binning average pupil dilation provides a convenient approximation to run repeated measures models with chosen option valence as a within-subjects factor. The results from the post-decision epoch clearly demonstrate a smaller magnitude of sustained pupil dilation for post-WoF win shapes [relative to post-WoF loss and post-WoF neutral shapes] when the reward probability of the chosen options is low, and a larger magnitude of sustained pupil dilation when the reward probability of chosen shapes is high. This crossover pattern of behavior closely resembled the crossover trajectory of the probability weighting function that captures participant choice behavior (Figure 3A, from low to high probability range). A repeated measures ANOVA indicated that, irrespective of AuC modality (i.e., whether it is behavior or pupil, F(1,37) = 0.127, p = 0.723), the reward probability of chosen options significantly (F(1,37) = 26.374, p <0 .001) and differentially (i.e., the interaction term, F(1,37) = 25.572, p <0 .001) modulated these indices. Furthermore, the AuC computed from the probability weighting functions (Figure 3A) correlated positively with the AuC computed from pupil dilation in the post-decision epoch (Figures 4B and 4C, r(36) = 0.362, p = 0.025 shown in Figure 5). Taken together, our results suggested that the extracts of the decision model not only correlate with pupil dilation prior to choice (Figure 4A), but also with the extent of sustained and relative pupil dilation to win shapes following choice (Figures 4B and 4C). For completeness, we also report the black pixel counts, which can be a proxy for shape-specific luminosity, for post-WoF win and loss fractals. The values for the post-WoF win fractals were (in the order of 90, 80, 20, 10%): [3103, 3325, 6448, 7005]; post-WoF loss fractals were (in the same order): [2298, 3944, 2714, 9201]. Although the loss 10% seems to be an unintended outlier from the time of conceptualizing the study, it is unlikely to confound the results to a great extent. Here, the higher number of black pixels in a fractal should lead to pupil constriction as a larger part of the screen (although small overall) would not be emitting light. For example, high probability post-WoF win shapes overall had approximately 80 black pixels more than high probability post-WoF loss shapes, but still lead to a much greater pupil dilation during the outcome presentation period (Figure 4C).

## Discussion

In this work, we investigated the mechanisms underlying value-based decision-making between reward memories formed through RL and under the affective influence of WoF outcomes (Figure 1). Our learning results suggested that discrete affective events do not influence overall learning performance (Figure 2), limiting the possibility that subsequent memory guided value-based preferences originate from the influence of affective events on learning. When we probed the global organization of experimentally induced affective memories (i.e., all cases where these memories were probed by randomly drawn options with not only equal but also different probabilities), our findings suggest that healthy volunteers recall high value shapes learned after a WoF win outcome with further augmented reward probabilities, globally indicating a positive bias (Figure 3). Although there were some differences in the execution of preference tests between the two studies, we observed this positive bias in value-based recall across the studies, which was significant in 2 out of 3 assessments. A model-based pupillometry analysis suggested that the expected value regressor from this decision model is encoded by the central arousal systems governing pupil size (Figure 4A). A complementary model-free pupil analysis suggested that pupil dilation differentiates between the valence of affective memories during a binary preference test (Figures 4B and 4C). In the post-decision epoch, pupil dilation was most sensitive to the reward values of positively valanced memories (i.e., shapes learned following a win outcome on the WoF), closely reflecting the crossover pattern of behavior from low to high probability shapes revealed by computational modeling (Figure 3A). Taken together, these results illustrate that human memory-guided value-based decision-making is influenced by earlier experiences of discrete affective events and engages the pupil-linked central arousal systems prior to and after the decision onset.

In these studies, we investigated the degree to which nonclinical participants display a positive bias in value based between affective and non-affective memories. Our reference points in designing this experiment were the previous work that utilized WoF paradigms in subclinical human populations^18^^,^^19^ and a rodent assay that assessed the impact of rapid versus traditional antidepressants on a single negative memory relative to a single control condition.^20^ In our experimental protocol, we probed a much larger pool of experimentally induced affective memories relative to earlier human work in order to achieve a broader coverage of the probability spectrum to aid computational modeling of affective memory recall. For example, in Study 2 there were 24 abstract stimuli that could be uniquely paired with 23 other stimuli during the preference test, resulting in a total grid space of 552 combinations. Inevitably, this large stimulus space made it difficult to capture the essence of human behavior through rmANOVA models and subsequent model-free comparisons. Computational modeling allowed us to look beyond individual model-free comparisons and harness the full value of a rich dataset. This approach revealed that winning in the WoF augmented the reward values of abstract stimuli during recall to a greater degree than losing in the WoF (Figure 3 all panels). Consequently, when we consider this complexity and the global organization of human affective memories, we think that an overarching and reasonably conservative interpretation of our results is that nonclinical volunteers are overall positively biased in their value-based choices between experimentally induced affective versus non-affective [reward] memories. This was confirmed in an additional regression model on the AuC metric that we report in Figure 3, after controlling for age, gender, WoF order effects, and depressive symptom scores (i.e., the Beck Depression Inventory scores reported in Table 1, the main effect of intercept t(112) = 2.458, p = 0.016). We observed this positive memory bias more strongly for shapes with higher reward probabilities. It is highly likely that this is due to our experimental design, in which paired fractals during RL blocks did not have independent probabilities, which would encourage more frequent sampling of the lower probability shape, but these were set to *p* versus *1-p*. In this scenario, after participants identified the higher probability shape, they would be more likely to continue sampling the higher probability shapes as it would increase their chances to bank more monetary rewards, meaning that these would also have stronger representations in memory relative to lower probability shapes. However, it is rather unlikely that our positive bias results can be explained by recency (shapes which are learned after the WoF and nearer in time to the preference test) or practice effects, considering that in our protocol we randomized (i) the order of WoF on different days (both Study 1 and 2), (ii) whether shape sets will be associated with post-WoF win or post-WoF loss blocks (to be more stringent in Study 2), (iii) the side of stimuli associated with post-win and post-loss WoF (in Study 1, this was no longer needed in Study 2). Our findings are in line with earlier studies, which suggested that repeatedly formed reward associations lead to positive memory biases,^22^^,^^23^ but we also demonstrate a computational mechanism that accounts for how discrete affective events can further contaminate value-based memory recall of previously learned reward associations. The nearest comparable study in the literature investigated how serotonin influence value-based recall between affective memories. Our model-free results are consistent with the placebo group (n = 34) from Michely et al., (2020). For example, they did not show evidence for an outright positive bias in their placebo group when only equal value comparisons were concerned (also see their Figure 3B). However, their limited coverage of the probability spectrum (i.e., only focusing on 70 vs. 30% reward contingencies), smaller sample size in the placebo group relative to our work and relying solely on the model-free assessment of preference bias meant that, probably, they were not able to slice this process further. Our implementation allowed us to propose a computational model for value-based decision-making between affective memories, that can capture the nonlinearity and positive bias that we were able to demonstrate here.

Our results demonstrate that value-based decision-making between affective memories engages the pupil-linked central arousal systems, with a negative correlation indicating that the pupil dilates more to chosen shapes with a lower expected value computed by the decision model (Figure 4A). This is in line with recent computational work which showed that expected values of chosen options are negatively correlated with pupil dilation,^24^ and with findings from other studies which demonstrate that pupil dilation prior to decision onset encodes value beliefs about to-be chosen options.^25^ In the context of our study, this pupillary signal is independent of the uncertainty in the probabilistic association between shapes and outcomes, because this kind of *irreducible* uncertainty peaks at 50% (i.e., maximum entropy). Prior to decision-onset, our complementary analyses suggested that participants spent comparable time evaluating post-WoF win and loss outcome shapes (Figure S11). This would be in line with the general null effects of WoF outcome valence on learning, i.e., the pre-decision stage during the preference test did not require participants additional time to evaluate certain fractals to come to a decision. After the decision onset, the physiological response to experimentally induced affective memories is explained by a differentially sustained pupil dilation, that is particularly sensitive for the reward probabilities of post-WoF win outcome shapes (Figure 4B). This sustained physiological response was also positively correlated with a model-based index of positive bias (Figure 5), suggesting that individuals who displayed a stronger preference for high probability shapes encoded after winning in the WoF, also had a stronger physiological response to these shapes after value-based decision-making (i.e., decision onset). Intriguingly, larger pupil dilation for low probability shapes learned after a WoF-loss outcome (Figure 4B), and generally for options with a lower expected value (Figure 4A) may be in line with the interpretation that pupil dilation indexes error-monitoring during value-based decision-making. Considering that pupil dilation is under the influence of a number of neurotransmitters such as norepinephrine,^26^^,^^27^ acetylcholine^28^ and serotonin,^16^ our current work may be useful for understanding the effects of traditional and rapid-acting psychotropic compounds on affective memories while acting on these systems. For example, a recent study suggested that pupil dilation in response to negative prediction errors (i.e., outcomes worse than expected) correlates with lower dorsal anterior cingulate choline levels and self-reported depressive symptoms in adolescents.^29^ In adult participants, a similar physiological signature for outcome monitoring that differentiates pupil dilation for negative and positive outcomes was observed in healthy volunteers, but not in individuals with remitted depression.^30^ These findings add to earlier^31^ and more recent literature which suggests differences in physiological response to affective images, for example viewing emotional faces, in patients with current and remitted depression.^32^ Taken together, our results are globally in line with the findings in healthy volunteers reported by these studies, and perhaps suggest that the experimental affective memory probe that we report in this article may have clinical utility in understanding how behavioral and physiological response to affective memories may be impaired by symptoms of depression. Although in the current work we focused on the mechanisms of affective memory recall in healthy volunteers instead of recruiting participants with respect to low and high depressive symptoms, this is a worthwhile direction that future studies should pursue. Recently, we reviewed the cognitive effects of ketamine, an anesthetic compound with rapid antidepressant properties at lower doses, on value-guided choice, which highlighted the potential involvement of brainstem neurotransmitter hubs such as raphe nuclei and locus coeruleus^33^ (i.e., regions involved in serotonin and norepinephrine release, respectively) and there is preliminary evidence suggesting that selective serotonin reuptake inhibitors induce a specific positive bias during value-based recall.^19^ These studies, along with our ongoing work investigating the cognitive effects of ketamine on memory processes described in this article, will bridge the translational gap between human and rodent work that investigated the affective memory effects of traditional versus rapid antidepressants.

### Limitations of the study

Finally, it is important to highlight some limitations of the current study with a view to informing future experimental design. Here, it may be important to highlight that the discrepancy between real-life versus experimental probes may be higher for negatively valenced memories, e.g., witnessing a traumatic event versus experiencing a large magnitude loss in WoF. Nevertheless, the WoF paradigm allows us to experimentally manipulate and control similar underlying process across participants. One limitation that we share with preliminary work in this area is that we cannot dissociate the effects of mood from arousal which may be associated with winning or losing in the WoF. This is a common limitation of existing literature, and future studies may record physiological markers such as pulse or galvanic skin response during memory encoding to dissociate the effects of mood from physiological arousal. Secondly, in some of our model-free analysis of the preference tests we were not able to fully dissociate the interaction between WoF effects and the influence of different probability levels. This could be achieved by randomizing the order of the learning blocks, which we omitted for the current study as it would also mean recruiting a larger sample size, which was not feasible in a within-subject study design. Future studies may consider designing preference tests in which all shapes were paired with every other shape only once (e.g., one-shot trials covering 24×23 = 552 combinations in Study 2) instead of relying more frequently on equal value comparisons (Figure S9). Although this approach may be more demanding for clinical research, it might be better for computational modeling and completely moving away from relying on individual model-free comparisons to develop mechanistic models of affective memory retrieval. Where it is feasible in a within-subjects design, it may also be worthwhile to test affective memory-guided decision-making processes across 3 assessment points, instead of two as in our Study 1 where we administered the preference tests at 24- and 48-h post-learning (Figure S7). This can allow for understanding how experimentally induced affective memories gradually transition to be stored in the long-term memory. This process remains an open question, particularly based on our findings from Study 1 which suggested that the differences between memories associated with positive and negative outcomes are still augmented between 24 and 48 h. Therefore, further assessments at 72 h or one week later could shed light on this question. Finally, it we would like to highlight that it may be worthwhile to conduct brief psychiatric assessments with potential study participants to rule out hidden psychiatric symptoms. Future studies should also consider exerting even tighter controls over their choice of abstract fractals in similar experimental studies, as potential [black] pixel count differences may constitute unintended confounds.

### Conclusion

In this work, we expanded on previous human^18^^,^^19^ and rodent^20^ studies, and provided a detailed account of the mechanisms underlying human RL and decision-making between experimentally induced affective versus non-affective memories and their corresponding physiology. Previously, this approach was used to understand the influence of traditional antidepressants on human memory retrieval,^19^ and in rodents as a test to compare the effects of traditional versus rapid-onset antidepressants.^20^ We propose that combining the pupillometry element from Study 1 with the balanced design of Study 2 that involved the full coverage of the probability spectrum during learning and decision-making, would have translational utility in bridging the gap between rodent and human models of affective memory recall.

## STAR★Methods

### Key resources table

**Table:** 

REAGENT or RESOURCE	SOURCE	IDENTIFIER
**Software and algorithms**		

MATLAB 2015a	MATLAB software	https://uk.mathworks.com/
Oxford Symbol Study	This paper	https://www.oxfordsymbol.com/
Behavioral learning and decision-making models	This paper	Data S1

### Resource availability

#### Lead contact

Further information and requests for resources should be directed to and will be fulfilled by the lead contact, Dr Erdem Pulcu (e-mail: erdem.pulcu@psych.ox.ac.uk).

#### Materials availability

This study did not generate new unique reagents.

#### Data and code availability

• Participant data underlying main figures are included as Data S1. All other anonymised behavioral data is available from the lead contact upon request.
• Behavioral modeling code is included as Data S1.
• Any additional information required to reanalyze the data reported in this paper is available from the lead contact upon request.

### Experimental model and study participant details

Forty-five (Study 1) and seventy-four (Study 2) English-speaking healthy participants were recruited from the general public using online and print advertisements around Oxfordshire, UK. Sample size was mainly determined by research feasibility concerns. Nevertheless both of the studies had much larger sample size relative to previous studies using a similar experimental design. All participants had normal or corrected to normal vision and did not report a present or past psychiatric diagnosis, nor any serious medical condition that could impact their study participation. Participants were excluded if they were currently using psychotropic medication. As with similar studies in healthy volunteers study exclusion was based on self-reports, and we did not conduct a formal psychiatric interview with potential participants. In Study 1, a single participant reported BDI scores above 13 (cut off point for minimal depression), whereas in Study 2 four individuals reported scores above this cut off point (max score 17). This subtle increase may be normal as Study 2 was conducted online during COVID-19 lockdown period. Participants received monetary reimbursement for their time (£50) plus additional payment depending on their task performance across the learning and decision-making components of the experiment (£33.26-£38.40, mean ± SD £37.25 ± 0.90). The study was approved by the University of Oxford Central Ethics Committee (CUREC; ethics approval reference: R66705/RE001). All participants completed an informed consent form conforming to the Declaration of Helsinki. Demographic information related to study cohorts (e.g., age, gender etc.) are available in Table 1.

### Method details

In general terms both studies used a within-subjects crossover design. In Study 1, testing sessions took place over 5 consecutive days at the University of Oxford, Department of Psychiatry at Warneford Hospital. On the first visit, the participants were taken through a screening interview to assess their eligibility. Then, the participants responded to a set of demographic questions and completed a battery of psychological questionnaires. After the screening interview, the eligible participants continued with the first day of learning and completed 3 blocks of a simple RL task to learn the associations between shape appearance and reward probabilities through trial and error (by selecting between left and right shapes on the screen using left and right arrow buttons). Participants’ affective state was manipulated using a WoF paradigm adapted from Eldar and Niv (2015).

To probe value-based recall of affective memories, after the training days, we asked participants to make decisions in a two-option forced-choice (TOFC) preference task in which various combinations of the abstract shapes they had learned about were paired with each other (i.e., on the last 2 days of the lab-based study, and the last day of the online study). Although no explicit feedback was given to participants in the preference test, they continued to accumulate money based on the reward probability of the chosen shape (i.e., 90% chance of winning 2p if the participant selects the shape associated with 90% reward probability in the learning phase). In the preference tests accumulated money was calculated but not displayed on-screen to ensure that participants cannot use this information as a proxy for their decision performance which would otherwise contaminate preference choice behavior with further learning. All tasks in Study 1 were presented on a laptop running MATLAB (MathWorks Inc) with Psychtoolbox (v3.1).

In Study 2, testing sessions took place over 3 consecutive days and were delivered using an online platform (due to the global COVID-19 pandemic). We manipulated the reward probabilities in each RL block pre-and-post WoF in a balanced manner to investigate how discrete affective events influence human RL. Further details of experimental procedures and statistical analysis approach are given below. All tasks in Study 2 were presented on a custom html/web-based platform designed specifically for this study (https://www.oxfordsymbol.com).

#### Training phase

In the lab-based study (Study 1) on each of the learning days, participants started with a baseline block (30 trials), followed by two more blocks (each 90 trials), with rest periods between the blocks. In the online study (Study 2), all blocks contained 40 trials each. The shapes in the RL tasks were selected from the Agathodaimon and Dingbat Cobogo fonts. During the RL blocks, participants were asked to learn the reward probabilities associated with each shape through trial and error. Different pairs of stimuli were presented in different RL blocks, so that different stimuli were presented both pre-and-post WoF and on different training days (apart from Study 1 where the baseline block was repeated identically on all 3 training days). For example, in Study 1 with win, loss and neutral WoF outcomes, the post-WoF 90% vs. 10% RL block on day 1 (WoF win outcome) would have shapes A and B, on day 2 (WoF loss outcome) shapes C and D, and finally on day 3 (WoF blank/neutral outcome) shapes E and F. Participants were explicitly informed that on any given trial if one shape is rewarded the other one is not rewarded (such that the probabilities are p versus 1-p). A green frame appeared around the rewarded option to provide feedback on participant choice. For every correct choice made, participants gained 2 pence and they were asked to accumulate as much reward as possible. Participants started with £15 on day 1 and a running total at the bottom of the screen was updated at the start of each subsequent trial.

In total, the participants were asked to learn reward probabilities associated with 14 shapes in the lab-based study and 24 shapes in the online study. The assignment of shape identities to different reward probabilities across pre-and-post WoF learning blocks was fixed in Study 1 but randomised in Study 2 to rule out any possible confounds as stringently as possible. Participant preferences between these shapes were later probed in the preference test which took place on the 4^th^ and 5^th^ days of the lab-based study and the 3^rd^/final day of the online study. All learning task blocks were self-paced and the sequence of rewarded vs. null outcomes within each RL block were pseudo-randomised and administered identically to each participant.

#### Wheel of fortune

Previous human studies suggested that Wheel of Fortune (WoF) paradigms involving large magnitude win and/or loss outcomes are useful probes for investigating the influence of discrete affective events on subsequent value-guided choice.^18^^,^^19^ To influence the participants’ affective state, a single WoF draw was used in the break between the first and second blocks on each of the learning days.

Participants were told that they could either win money, lose money, or receive nothing (blank). Unknown to the participants, the draw was not random but fixed, such that in the lab-based study each participant won (+£15), lost (-£11), or received a blank outcome (£0) across the 3 days (see Figure 1 and legends about a detailed description of the experiment). In the online study, we excluded the blank condition and focused only on win (+£14) and loss (-£7) outcomes, and adjusted their outcome magnitudes considering the commonly observed salience differences between wins and losses.^34^ To control for an effect of order, these outcomes were counterbalanced across participants. In Eldar and Niv^35^ (2015), individual trials were rewarded with 25 cents and participants could either experience a loss or win of $7 after a single WoF draw (1/28 ratio). In contrast, we decided to increase the WoF outcome/reward ratio to ensure that these affective events (i.e., WoF outcomes) would feel more salient to participants. Therefore, in our experimental design, each correct prediction during RL led to a smaller reward (2p). As illustrated in the WoF outcome slices shown in Figure 1A, win and loss outcomes were sandwiched between large magnitudes of opposing outcomes, creating a near-miss effect aimed at strengthening the affective impact of the WoF. We used these large magnitude WoF outcomes to experimentally induce negative or positive memory biases. Using this design, we explored whether discrete affective events, here induced by winning or losing in a WoF draw, influence immediate performance in reward learning or induce learning biases. Recent RL studies have demonstrated that negative or positive learning biases may develop even in healthy volunteers as a rational response to environmental contingencies^36^ and relate to poor filtering of informative negative experiences from uninformative ones.^37^

#### Happiness ratings

During the learning days, participants were asked to report their current happiness. In the lab-based study (Study 1), we used a Likert scale from 1 to 9 (with higher numbers indicating greater happiness), whereas in the online study (Study 2) we used a visual analogue scale. Participants were asked to provide a happiness rating at different time points during the experiment, for example before starting the first RL block, more critically immediately before and after the WoF, and at the end of their training on any given day. All the design features of the studies are summarised in Table 2 below.

#### Preference tests

In the last phase of the experiment, we conducted preference tests. In order to assess the test-retest reliability of participant preferences we conducted the preference test twice, once on the 4^th^ day and again on the 5^th^ day in the lab-based study. Participants were presented with random pairs of all shapes they encountered during the training days and on each trial, were asked to choose the shape with the higher reward probability. Participants continued accumulating monetary rewards (2 pence per trial) in relation to the reward probability of the chosen shapes. For example, if the preference trial involves 90% win probability versus 20% win probability shape and if the participant correctly identifies the 90% probability shape, they would have 90% chance to gain 2p in that trial. If the lower probability (i.e., 20%) shape was chosen, they would have 20% chance to gain 2p in the preference trial. These probabilities were drawn online during the preference test. Consequently, we refer to this process as value-based recall in the main body of the article and our aim was to examine whether a WoF induced change of affective state would bias their valuations of the learned shapes. Written and spoken instructions were given and subjects were presented with a print-out of all the shapes (14 in Study 1 (lab) and 24 in Study 2 (online)) before the task began, to provide them with an opportunity to recall the learned shapes. Within each study, shapes were randomly paired with each other to cover all possible combinations leading to 400/456 trials (lab-based versus online) presented across three blocks, and the order of these trials was randomised across participants. We were particularly interested in the pairs of shapes that had objectively identical reward probabilities but appeared after different WoF outcomes, thus should be encoded under different affective influence (Figure 1). Therefore, the majority of trials presented during the preference tests prioritised randomly pairing equal probability shapes such that the absolute value difference between the options would be near 0 (Figure S9).

In the preference tests (both Study 1 and 2), participants did not receive explicit feedback about whether their choices were correct, so they had to rely on what they had previously learned. To prevent further learning, participants' running total did not appear on the screen during a block of trials, but they did continue to accumulate money based on whether their choice was correct and the reward probability of the chosen shape. Their running total was only displayed between blocks to provide an indication of their performance in the previous block. The trials in the preference tests were also self-paced and these took roughly 30 min for the online study and 50 min for the in-lab study with pupillometry. Using this experimental design, we investigated whether experimentally induced changes in emotional state influence human memory-guided value-based decision-making 24 and/or 48 h later.

In the preference tests of Study 1, the pre-WoF/baseline shapes (70% vs. 30%) and post-WoF neutral outcome shapes were presented on both sides of the screen, whereas the post-WoF win and loss outcome shapes were presented exclusively only on one side. This sidedness in post-WoF win and loss stimuli presentation was counterbalanced across participants and between preference test days 1 and 2. The reason we took this approach was to cover as many discrete values in the reward probability spectrum (particularly the mid-points at 70% and 30%) with the least number of abstract fractals. When designing Study 1, we were unsure whether participants would adequately remember more than 14 shapes and their probabilistic relationship with reward. Furthermore, this approach enabled the estimate of the probability weighting function by providing additional points to fit along the probability spectrum (i.e., 10, 20, 30, 70, 80, 90%). Conceptually, this implementation would also be in line with one definition of affective bias, that is, how affective content can influence otherwise non-affective and neutral content. However, we revised this approach in conceptualising the Study 2 to increase the stringency of our results. This also reflected the qualitative feedback from participants who indicated that it was feasible to remember 14 shapes. In Study 2, the total number of shapes therefore increased to 24.

#### Questionnaire measures

In addition to the computerised task, participants were asked to respond to a series of self-report questionnaires on the first day of their testing, prior to completion of the first learning block. These questionnaires included: (1) Beck Depression Inventory (BDI-II),^38^ a standard measure of depression; (2) Spielberger State-Trait Anxiety Inventory (STAI),^39^ an anxiety measure comprising trait (anxiety proneness) and state (current state of anxiety) subscales; (3) Positive and Negative Affect Schedule (PANAS), assessing the feeling and expression of positive and negative emotions^40^; (4) Behavioral Activation/Behavioral Inhibition (BIS/BAS), reflecting aversive motivation and appetitive motivation^41^; (5) and the Mood Disorder Questionnaire (MDQ),^42^ a screening tool for Bipolar Disorder.

### Quantification and statistical analysis

We used repeated-measures analysis of variance (rmANOVA) models to investigate the effects of our experimental manipulations. All significant main effects and interaction terms were followed up by post hoc tests, corrected for multiple comparisons using Bonferroni correction. All analyses were conducted on MATLAB and SPSS v29. In the lab-based study, 6 participants were excluded from the analyses of pupillometry data due to signal dropout affecting more than half of the trials, and in total 2 participants were excluded from the behavioral analysis (one person reported intentionally selecting the lower probability shapes during the training phase, the other person dropped out from the study before preference test 2). These 2 individuals were among the 6 individuals excluded from the pupillometry analyses. In total, 5 participants were excluded from the online study, 4 individuals who did not give more than 4 unique mood ratings across 16 assessment points, and one individual who consistently selected the lower probability shapes more frequently than the higher probability shapes. Across all statistical models, reward probability, valence and behavior in pre-post-WoF blocks were entered as within subject factors, whereas WoF order (e.g., whether participants experienced a win or a loss outcome on the 1^st^ day) and shape set which randomised the associations between shape identities and reward probabilities were entered as between subject factors. We further analyzed participant choice behavior by computational modeling that relies on the probability weighting function (see below and refer to the supplemental information for further details).

#### Use of probability weighting function for logistic regression/computational models

In the current study, we analyzed participant choice behavior with a well-established computational model of value-guided choice, which posits that choice preference between binary options with different reward probabilities can be expressed in terms of weighted probabilities.^43^ We predicted that affective response associated with winning or losing in the WoF will contaminate how participants will recall learned reward probabilities in the subsequent preference test, for example winning in the WoF may increase the subjective reward probability of post-WoF shapes, whereas losing in the WoF may decrease the subjective reward probability of these shapes. In practical terms, this would mean that following a large magnitude win outcome in the WoF, a shape associated with 80% objective reward probability could be recalled and subjectively recalled as 85% or even higher due to the effect of the positive WoF outcome (i.e., learning those reward probabilities under the influence of positive mood created by winning in the WoF). Our proposal is in line with recent theories of episodic RL^44^ and formalises the potential nonlinear effect of discrete affective events through the use of probability weighting function during retrieval. We predicted that WoF outcomes will have an influence on subsequent memory-guided value-based decision-making^45^ and that the influence of affective events will be prominent for high probability shapes as they should be selected more frequently during the learning/encoding phase. By relying on the 2-parameter exponential-logarithmic probability weighting function, we aimed to describe a model that accounts for participants’ subjective probability estimates during value-based recall in an unbiased manner. Recently we have demonstrated the versatility of this approach when applied to other cognitive domains, for example affective biases in human facial emotion recognition.^46^ Using this model, we tested an *a priori* hypothesis that a non-clinical population would overall display a preference for shapes with higher reward probabilities that is further augmented by winning on the WoF. Note that the parameters of the probability weighting function are estimated directly from participants binary choices, but not from summary statistics of choice behavior (e.g., Table S1). In Study 1, the parameters of the model were fitted to participant choices in the preference test in a way that accounts for how the probabilities of all shapes presented on the same side as the post-WoF win shapes or the same side as the post-WoF loss shapes may be distorted (the side was counterbalanced across participants and between two preference day assessments to rule out any confounds). Therefore, in Study 1 the model predicts whether the participant will choose the left (e.g., post-WoF win shapes side, with its own delta and gamma parameter) or the right (e.g., post-WoF loss shapes side with its own delta and gamma parameter) option (with an additional inverse temperature term for choice stochasticity). In Study 2, the parameters were separately fitted to the post-WoF win and post-WoF loss outcome shapes as the preference test did not have any sidedness in stimuli presentation and all shapes were presented on different sides of the screen randomly. Both studies revealed qualitatively overlapping results, particularly for high probability shapes where we expected the effects to be more prominent due to stronger encoding during the learning phase.

#### Pupillometry

On the first day of preference tests (day 4) in the lab-based study, we collected pupillometry data to assess physiological response to affective memories during value-based decision-making. We used pupillometry to extend our recent findings which demonstrated that the information content of negative affective events engages the pupil-linked central arousal systems.^36^ We investigated whether value-based recall of affective memories also engages central arousal systems. By using the regressors generated by the behavioral model that accounts for participant preferences, it is possible to identify specific aspects of the choice behavior that are encoded by the central arousal systems, lending physiological support for model components (similar to analysis of model-based fMRI^47^^,^^48^). We used a multiple linear regression model to explain the physiological response immediately before, during, and immediately after making choices between shapes learned following different WoF outcomes. In line with our previous pupillometry work,^36^^,^^46^ we used a model-based approach in our analysis of pupillary data and tested the prediction that subjective values that guide decision-making between affective memories will significantly influence pupil dilation. We performed complementary analyses of the sustained pupil dilation to investigate whether discrete affective events that were experienced up to 72 h before, continue evoking differential arousal response during value-guided choice. These 2^nd^-level repeated measures ANOVA models were implemented on either regression coefficients from the 1^st^-level (Figure 4A) or on raw pupil dilation averaged at every 1 s bin (Figures 4B and 4C).

During pupillometry recording participants' heads were placed at 70 cm distance from the computer screen, stabilised by a chin rest. The eye-tracking system (Eyelink 1000 Plus; SR Research, Ottawa, Canada) was linked to the presentation computer through an ethernet connection. The sampling rate for pupillometry was set to 500 Hz recording from both eyes. The preference test was presented on a VGA monitor. A fixation cross marking the middle of the screen separated the presented pair of shapes.

Preprocessing of the pupillary data involved removing eye blinks that were identified using the built-in filter of the Eyelink system. A linear interpolation was implemented for all missing data points (including blinks). The resulting pupil trace was processed through a low pass Butterworth filter (cut-off of 3.75 Hz) and then z-transformed across the preference test session.^49^^,^^50^ In order to assess phasic response to task-related variables, we performed baseline correction by subtracting the mean pupil size during the 2-s baseline period prior to our epochs of interest (i.e., decision and outcome) from each time point in the post-stimuli presentation period. Individual trials were excluded from the pupillometry analysis if more than 50% of the data from the outcome period had been interpolated.^36^^,^^49^ The preprocessing resulted in a single set of pupil time-series per participant containing pupil dilation data for each of the included trials.

## Supplemental information

Document S1. Figures S1–S11 and Tables S1–S3

Data S1. Numerical estimates underlying main figures
