# Supplementary material for: Value-based decision-making between affective and non-affective memories
Source: iScience. 2024 Feb 24;27(3):109329. doi: 10.1016/j.isci.2024.109329 (PMC10933545; doi:10.1016/j.isci.2024.109329)
Supplement: Document S1. Figures S1–S11 and Tables S1–S3 [file mmc1.pdf]

iScience, Volume *27*

Supplemental information

Value-based decision-making

between affective and non-affective memories

Erdem Pulcu, Calum Guinea, Hannah Clemens, Catherine J. Harmer, and Susannah E. Murphy

Supplemental Information

Modelling reinforcement learning. Participants' choice behaviour during the learning phase can be captured by a simple reinforcement learning model. For each participant, a learning rate (α) was estimated separately for each of the most rewarded shapes in each block of learning trials (e.g. a learning rate for the 90% reward probability shape which appeared immediately after losing on the WoF), using a Rescorla-Wagner learning rule which updates prediction errors (PEs) with a learning rate parameter[1]:

$$p_{(i+1)} = p_{(i)} + \alpha(Out_{(i)} - p_{(i)}) \qquad (1)$$

In this equation, $p_{(i)}$ is the estimated reward probability associated with shape A, and $Out_{(i)}$ depicts whether shape A was rewarded or not on trial *i* (i.e. a vector of ones and zeroes where 1 indicates that the reward was associated with shape A). Considering that the reward probabilities were interdependent within each shape pair, the probability that shape B is rewarded on the current trial is 1-$p_{(i)}$. In Equation 1 α is the learning rate estimated from participants' choices.

We analysed participant choice behaviour with 4 variants of this model. All models had 3 free parameters in total, estimated individually from each participant's choice behaviour.

Model 1 had two learning rates, updating participant's belief about the probability of the better shape depending on the sign of the prediction error (i.e. learning rate for positive prediction errors and one learning rate for negative prediction errors).

Model 2 was based on a dynamic learning model recently reported as the best fitting model in another study investigating the effect of WoF outcomes on human learning and memory[2]:

$$\alpha = \frac{1}{\varepsilon + t_1^{40}} \qquad (2)$$

where $\varepsilon > 0$ is a free parameter individually fitted to each participant data and additive trial numbers (*t*) in the denominator allow learning rates to diminish towards the end of the block, which is a widely accepted assumption for learning stable contingencies. The notation t^{40} is

because each RL block in the online study (Study 2) contained 40 trials. In this model, the WoF outcomes influence the subjective value of the rewards received during learning:

$$O\tilde{u}t_{(i)} = Out_{(i)} + \theta \quad (3)$$

where actual outcomes are transformed by an additive free parameter (θ), estimated between bounds [-2.5,2.5].

Model 3 had a single learning rate as shown in Eq.1. This model assumed that participants' mood would influence RL on a trial-by-trial basis. Each participant's mood trajectory within a block was retained by linearly interpolating between participant mood ratings which were captured by a visual analogue scale (VAS) at the beginning and the end each learning block (ratings as shown in **Figure S1).** The assumption tested by this model was that participants' current mood would make it easier or more difficult to identify the better shape during the learning block. The effect of participant mood is implemented at the choice level, modulating decision values ($\Delta\tilde{v}$) in a sigmoid function which generates choice probabilities for the better shape (q_A):

$$\Delta\tilde{v} = p_1^t - (1 - p_1^t) \quad (4)$$

$$q_A = 1/(1 + e^{(-\beta(\Delta\tilde{v} + \omega\mathbb{Z}_1^t))}) \quad (5)$$

where ω is a free parameter estimated in the same space as θ in Eq.3, and $\mathbb{Z}_1^t$ is the vector of interpolated mood ratings, and $\beta > 0$ is an inverse temperature parameter to capture the stochasticity in participant choices.

Finally, in Model 4 we tested whether separate learning rates assigned to update small and large magnitude of PEs can better account for participant choice behaviour during RL. A recent study demonstrated, within the context of Pavlovian conditioning, that experience of large PEs create event boundaries in human memory such that sequence of stimuli across a high PE event are recalled less accurately[3]. This model had the same 2 learning rate structure of Model

1, with an additional free parameter (0< $\kappa < 1$) that serves as an individual sensitivity threshold to categorise trial-wise PEs into small versus large for each participant, and participant choice probabilities are generated by a sigmoid function driven by the value difference between the shapes (as in Eqs. 4 and 5).

In order to choose between these models we relied on the group-wise sum of Bayesian Information Criterion (BIC), which is a widely used metric for model selection[4] (**Figure S4)**.

Modelling participant choice behaviour in the preference tests. In this study, our primary research question is related to understanding value-based decision-making between affective memories, i.e. information encoded immediately after affective events such as winning or losing in a WoF draw. Considering that reward probabilities in RL blocks following the WoF draw were identical on each of the training days (e.g. 90% win probability in all RL blocks immediately after the WoF), a preference for one type of shape when participants are asked to choose between equal probability shapes can be understood in terms of probability weighting. This is because, when equal probability shapes are pitted against each other (e.g. a preference test trial in which participants are asked to choose between Win 90% versus Loss 90% shapes), preference for one type of shape means that either the reward probability associated with that shape is over-weighted, or the reward probability associated with the less preferred shape is under-weighted. This suggests that the 2-parameter probability weighting function[5] can adequately capture participants' preferences for shapes learned in different affective states. The strength of this approach is that the 2-parameter probability weighting function is free of any *a priori* assumptions about the shape of biases in probability weighting and can account for any non-linear weighting trajectories across the probability spectrum (i.e. between 0 and 1).

In the lab-based study (Study 1), we had abstract shapes falling into 4 different categories: baseline (i.e. pre-WoF shapes), win, loss, and blank depending on the outcome of the WoF on a given training day. This means that the ideal choice model guiding participants decisions can be expressed in terms of 8 parameters (2 parameters per shape category), accounting for

probability weighting for each shape category. However, particularly in the baseline condition, our experimental design for the lab-based study did not allow enough coverage of the probability spectrum as the baseline condition only involved shapes with reward probabilities 70% versus 30%. These limitations, arising mainly from feasibility issues (i.e. limiting the number of shapes participants were asked to remember), meant that fitting a 2-parameter non-linear function to 2 data points on the probability spectrum (i.e. at 30% and 70%) is likely to give unreliable parameter estimates. To overcome this limitation, during preference testing we only presented win shapes on the left side and loss shapes on the right side, while blank and baseline shapes were presented on each side randomly. This side-specific stimuli presentation was counterbalanced across the participants and between preference test days 1 and 2. This approach allowed us to use the baseline shapes to increase the coverage of the probability spectrum at the data points which were otherwise missing in win and loss conditions, i.e. both conditions only had shapes associated with reward probabilities 10%, 20%, 80%, and 90%. Secondly, by presenting the blank outcome shapes randomly on each side we increased the number of trials with which we can assess the impact of affective events on low (i.e. 10% and 20%) and high (i.e. 80% and 90%) probability shapes. This approach allowed us to capture the degree to which negative and positive events contaminate otherwise neutral information (one widely accepted definition of affective bias) during value-based decision-making and to model participant choice behaviour in the preference test.

In the online study (Study 2), we did not have the blank/neutral condition and the reward probabilities associated with the shapes covered the probability spectrum more evenly (**Figure 1A**) which meant that we were able to present all shapes randomly on either side, overcoming some of the design limitations of Study 1.

We used the probability weighting function to estimate perceived probabilities associated with shapes presented on each side:

$$\tilde{p}_L = e^{(-\gamma(-\ln(p_L)^{\delta})} \quad (6)$$

Where p_L is the estimated probability of reward associated with any given shape, and it is based on participants' learning behaviour linking the different stages of the experiment. The values of free parameters γ and δ were estimated between 0 and 4.5, as setting up higher numbers for the upper boundary of these parameters has limited effect on the non-linear trajectories of the weighted probabilities. Then, the choice model assumes that participants will make decisions based on the expected value difference between available options presented on the left and the right side:

$$\Delta\tilde{v} = \tilde{p}_L - \tilde{p}_R \quad (7)$$

The trial-wise stochastic choice probabilities of each shape were generated using a sigmoid function[6].

$$q_L = 1/(1+e^{(-\beta\Delta\tilde{v})}) \quad (8)$$

where $\beta > 0$ is the inverse temperature parameter, that governs the degree of stochasticity in participant choices. Values of β tending towards 0 reflect an increase in stochasticity, whilst values of β tending towards ∞ reflect more deterministic choices.

All free-parameters in computational models were estimated using a Bayesian model fitting procedure implemented in MATLAB. More specifically, the parameters were estimated by computing the full joint posterior probability of the models over the whole parameter space, and exact values of the free-parameters were computed by integrating the marginalised probability distributions of each parameter within specified parameter boundaries. Where applicable, learning rates were estimated in the inverse logistic space, probability weighting parameters were estimated in the normal space, and inverse temperature parameters were estimated in the log space. These are identical to parameter estimation procedures reported in our previous studies[7,8].

Supplementary Results

Effect of Wheel of Fortune outcomes on happiness ratings

In the lab-based study (Study 1), there was a significant main effect of WoF outcome on happiness ($F(2,64) = 9.388$, $p < .001$) and a significant WoF outcome by assessment time point interaction (i.e. the time point in which participants rated their momentarily happiness, $F(2,64) = 33.467$, $p < .001$). Importantly, there was no main effect of training order (e.g. whether participants experienced the win or the loss outcome on the WoF draw, $F(5,32) = .964$, $p = .455$) on any of the happiness ratings. As predicted, participants' self-reported happiness ratings were significantly lower after losing the WoF draw ($t = 5.59$, $p < .001$, pairwise comparison of pre-WoF and post-WoF on loss day), whereas they were significantly higher on a WoF win day ($t = -8.53$, $p < .001$, pre-WoF vs. post-WoF, win day; **Figure S1**). These effects on happiness were maintained to the end of the final learning block on the loss day ($t = 2.1$, $p < .05$, comparing happiness pre-WoF to happiness at the end of the experiment on the loss day) but not the win day ($t = -1.75$, $p = .088$, comparing happiness pre-WoF to happiness at the end of the experiment on the win day), indicating that both winning and losing on the WoF induced significant changes in current happiness but that the effects of losing on the WoF had a longer-lasting impact on happiness than winning. The mood ratings assessed at the end of each day were significantly different between the conditions ($F(2,134)=3.45$, $p=0.035$). Pairwise comparisons indicated that happiness ratings were significantly lower at the end of the loss day relative to the win day ($t(88)=2.7231$, $p=.008$). Comparisons against the blank WoF were not significant ($t(88)=1.5027$, $p=.137$).

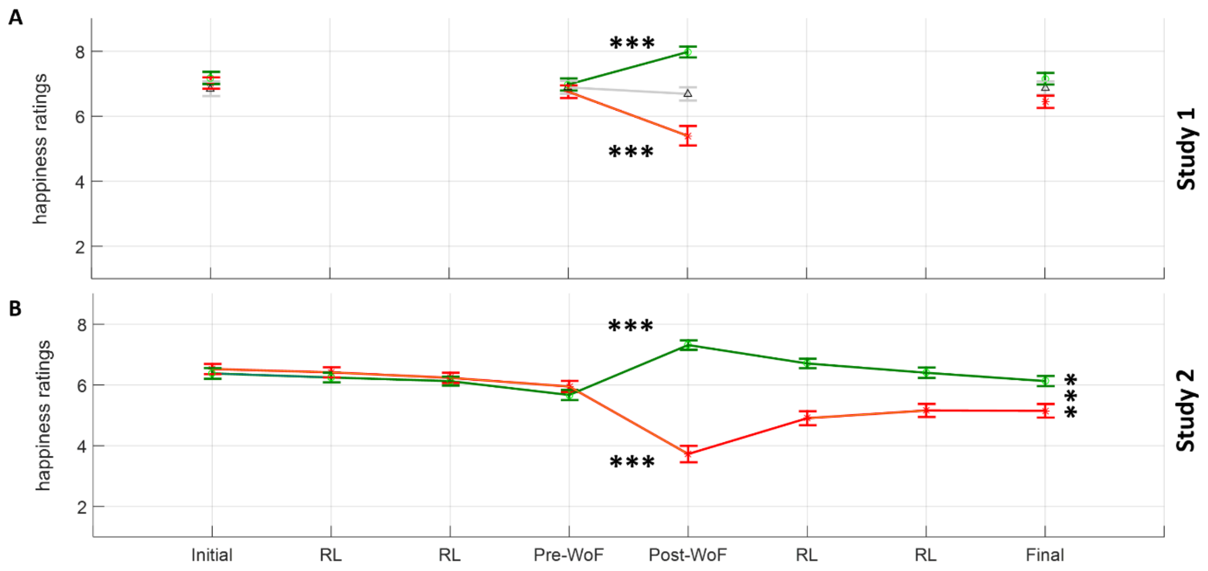

Figure S1. **Change in happiness ratings in response to Wheel of Fortune outcome, related to STAR Methods. A.)** In the lab-based study, participants' current happiness significantly improved post-WoF on a win day (green line), while significantly worsening on a loss day (red line). On the day with a blank WoF draw (grey line), there was no significant difference between pre- and post-WoF happiness ratings. Moreover, initial and final happiness ratings were comparable on all but the loss day. On the day with the loss outcome, although current happiness recovered significantly from post-WoF, it was still significantly lower than participants' initial rating. **B.)** In Study 2, mood was sampled more frequently than Study 1 (also see Table 2, blue diamonds for direct comparison between the studies). The findings related to mood change were replicated in the [online] Study 2. The green line indicates mood ratings on the training day with a win on the WoF, whereas the red line indicates the mood ratings on the loss day. Across all panels, error bars reflect ±1 SEM. ***p<.001. RL blk: reinforcement learning block. In Study 2, the happiness ratings were made at the end of each block, apart from the initial rating which was made before participants started their daily training session.

In the online study (Study 2), we mostly replicated these effects in a complementary rmANOVA model which suggested a significant main effect of WoF on happiness ratings ($F(1, 67)= 5.431$, $p = .023$), with a significant main effect of WoF outcome valence (i.e. win or a loss, $F(1, 67)= 96.120$, $p<.001$) and a significant valence by WoF interaction term ($F(1, 67)= 133.155$, $p<.001$). Again, there was no main effect of training order on the happiness ratings ($F(1, 67)= 2.661$, $p = .108$). Although participants mood ratings recovered from their lowest point which was immediately after a loss on the WoF, these still remained significantly lower on the loss day relative to the win day, even at the end of the experiment ($t(136) = 3.675$, $p<.001$, **Figure S1**). In Study 1 only a single participant reported higher mood rating following a WoF-loss outcome. There was no mismatch for WoF-win outcome and higher mood ratings. In Study 2, one individual reported a lower mood rating following a WoF-win outcome, and four individuals reported higher mood following a WoF-loss outcome. These were unique participants (i.e. no same participant making ratings consistently in the unexpected direction which may warrant

an outright exclusion). In Study 2, we had used a visual analogue scale to increase the precision of mood ratings where the participants can click on a precise location on a continuum (instead of a Likert scale in Study 1) and in the wide majority of these cases the mismatched ratings were in the decimal place (for example, rating 3.87 before the WoF draw and 3.96 after a WoF-loss outcome). These individuals were not excluded from the analyses.

Exploratory analyses of the effect of discrete affective events on human reinforcement learning

In Study 1, we were only able to partially investigate whether the valence of discrete affective events (i.e. winning, losing or a blank outcome on the WoF) influence human reinforcement learning. A rmANOVA did not reveal any significant main effect of WoF outcome on the probability of choosing the better shape (i.e. the shape associated with higher reward probability) post-WoF. This is illustrated in **Figure 2A**, where we show that valence of the WoF outcome does not influence learning in the post-WoF blocks. Moreover, the interaction term (WoF outcome by reward probability) was not significant and there was no main effect of WoF outcome order (all $p > .136$). However, in this study (Study 1) we were only able to compare learning behaviour in the post-WoF blocks which were identical in terms of their reward probabilities, but we were not able to understand how learning behaviour might have changed from the pre-WoF baseline, as the reward probability in the pre-WoF block was different (i.e. 70%) and the same baseline block was repeated across all 3 learning days in an effort to reduce the number of shapes that participants need to learn. We addressed this question by improving on the experimental design in Study 2 in which participants completed an identical number of blocks pre-and post-WoF with identical reward probabilities (**Table 2**). As stated in the main body of the article, these design changes did not lead to a WoF outcome valence effect on participant learning behaviour in the post-WoF blocks. Further model-free learning curves and participant’s choice frequency in the last 10 trials at different reward probability levels are shown in **Figures S2 and S3**.

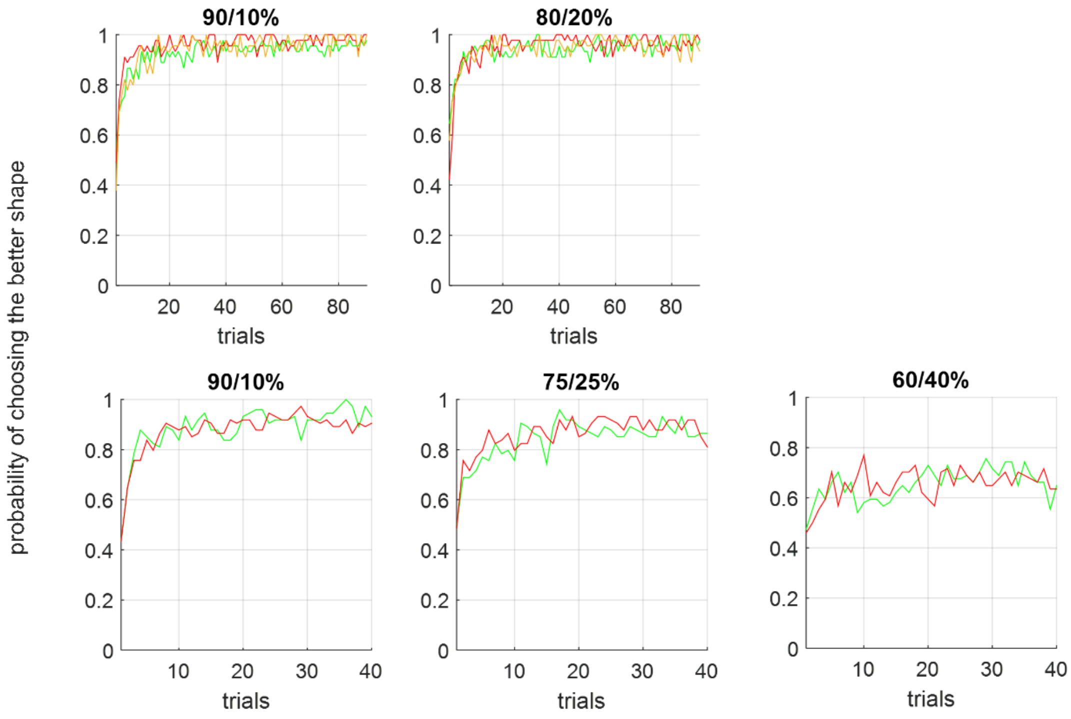

Figure S2. **Learning curves in post-WoF RL blocks, related to Figure 2.** Across both studies learning curves were globally overlapping confirming a null effect of WoF outcome valence on subsequent learning behaviour. Top row: Study 1; bottom row: Study 2. Green lines: learning behaviour after a WoF win outcome, red: WoF loss outcome; orange: WoF blank/neutral outcome (only in Study 1). Error shading not shown for ease of viewing.

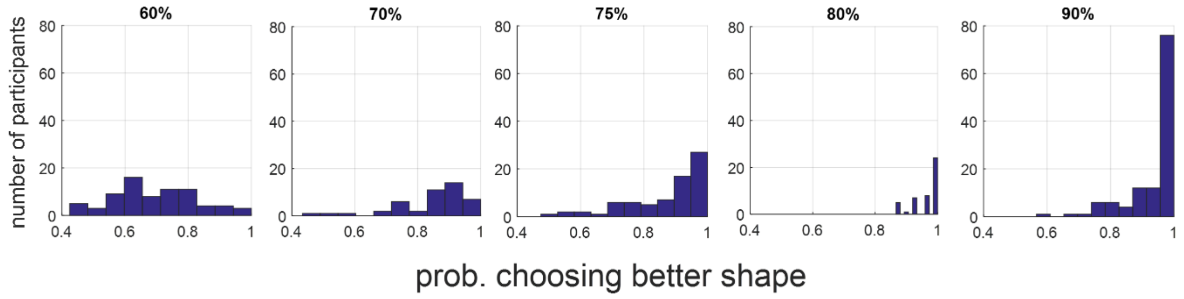

Figure S3. **Histograms of raw choice probability in the last 10 trials at each distinct reward probability level used across both studies, related to Figure 2.** Where applicable RL blocks for at a given reward probability level are clustered to show the overall picture. 70/30% learning block (2nd panel) only appeared pre-WoF in Study 1. 90/10% learning block was the only overlapping RL block between the studies (hence histograms are longer than other reward probability levels as shown in the rightmost panel**).**

We have committed to use the design of the Study 2 (this time also including pupillometry which was not possible for the current manuscript due to COVID19 restrictions at the time of data collection (April 2020)) in our ongoing work which aims to investigate the cognitive effects of ketamine as the Study 2 had a well-balanced design (clinicaltrials.gov identifier: NCT04850911). Due to lack of a significant main effect of WoF on learning behaviour in the

blocks subsequent to it, we did not conduct further exploratory analyses of the data from Study 1 with computational models.

Model-based analysis of reinforcement learning

In Study 2, where the reward probabilities of pre-and-post WoF learning blocks were matched, there was a significant main effect of WoF phase (i.e. pre versus post-WoF, $F(1,65) = 17.423$, $p<.001$), reflecting an increased tendency for participants to select the shapes associated with a high probability of reward in post-WoF blocks (**Figure 2B**). In order to understand mechanisms underlying improved learning performance in the post-WoF blocks from pre-WoF performance levels irrespective of WoF outcome valence (i.e. post-WoF phase effect, as reported in the main body of the article, see the change in height of bars pre-and-post WoF in **Figure 2B**) we further analysed participant choice behaviour in the online study using computational modelling. We were interested in exploring whether there could be any valence specific influence of the WoF outcome on common reinforcement learning model parameters such as learning rates or choice stochasticity.

We modelled participant choice behaviour by fitting four different computational models (see Modelling Supplement for details). A well-established model selection method based on computing Bayesian Information Criterion (BIC) values indicated that participants learned from rewarded and unrewarded outcomes (i.e. negative and positive prediction errors) with separate learning rates and made decisions between the shapes stochastically based on the expected value difference between the higher and lower probability shapes (i.e. Model 1, **Figure S4**).

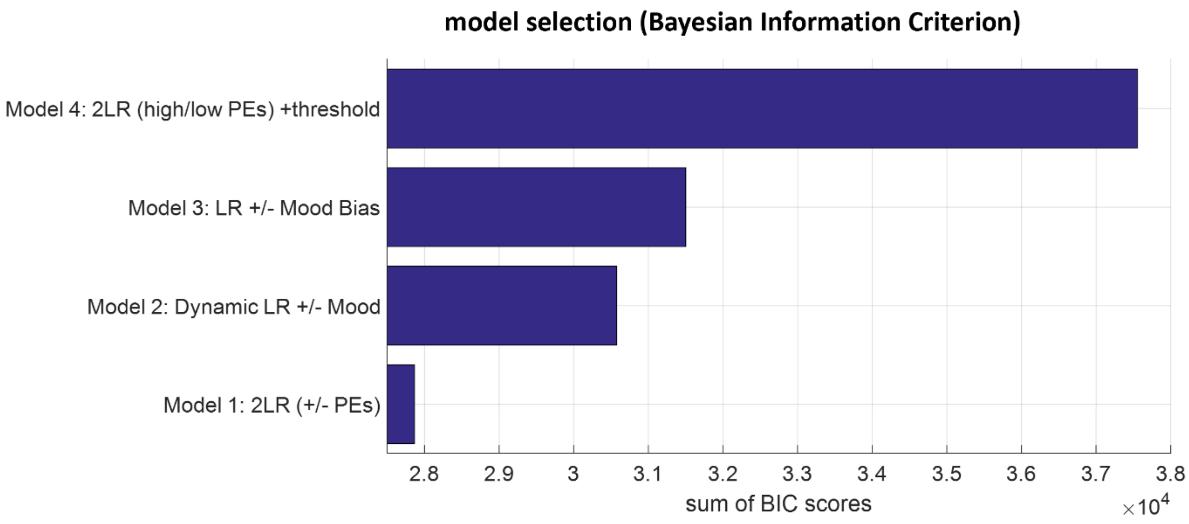

Figure S4. Best fitting computational model that explains participant's learning behaviour, related to Figure 2. Model selection based on group-wise sum of BIC scores comparing reinforcement learning models in the online study in which a model-free behavioural analysis based on participants' frequency of choosing the better probability shape (in Figure 3B) suggested a significant main effect of WoF on human reinforcement learning. The best fitting model (Model 1) suggest that participants update their reward probability estimates by 2 distinct learning rates for negative and positive prediction errors.

We fitted a 2x3x2 rmANOVA model to positive learning rates (i.e. learning rate updating positive PEs arising from RL feedback). This analysis indicated a marginally significant main effect of WoF ($F(1,65) = 3.904$, $p = .052$), reflecting higher positive learning rates in the post-WoF blocks but no main effect of valence ($F(1,65) = .117$, $p = .733$, **Figure S5A**). There were no significant interactions between these factors and no main effect of WoF order ($F(1,65) = .094$, $p = .760$). Analysing the negative learning rates in the same manner indicated a significant main effect of WoF phase ($F(1,65) = 4.328$, $p = .041$). There was also a significant main effect of outcome reward probability ($F(1,65) =16.277$ $p<.001$) and a marginally significant main effect of WoF outcome valence ($F(1,65) = 3.068$, p = . 085, **Figure S5B**). Although there was no 3-way interaction, and no main effect of WoF order ($F(1,65) = .422$, $p = .518$), there was a significant interaction between outcome reward probability and WoF outcome valence ($F(2,130)= 4.066$, $p = .019$). Losing in the WoF reduced learning rates from negative prediction errors in response to RL feedback in blocks associated with higher expected uncertainty (i.e. reward probabilities 75% and 60%). Finally, repeating the same analysis for the inverse temperature term which governs choice stochasticity during RL

indicated a significant main effect of WoF ($F(1,65) = 7.503$, $p = .008$) and a significant main effect of outcome reward probability ($F(2, 130) = 75.499$, $p<.001$), with no main effect of WoF outcome valence ($F(1,65) = 2.162$, $p = .146$, **Figure S5C**). There were no significant interaction terms between these main effects and no main effect of WoF order ($F(1,65) = 2.286$, $p = .135$). Overall, inverse temperature estimates were higher in the post-WoF blocks suggesting that participants were less stochastic in their choice behaviour. One might argue that this finding may be an artefact of practice effects, i.e. participants performing the task better in the post-WoF blocks. However, in Study 2 where participants completed 3 blocks of RL before the WoF, this kind of higher order learning about the structure of the task should also have happened before the WoF (e.g. at least by the beginning of 3rd block instead of specifically on the 4th block which is immediately after the WoF). Secondly, there may be limited reasons to suspect that after such higher-order learning, the behavioural performance should fall to a baseline the next day and to improve yet again in the post-WoF blocks). The results from a complementary model-free analysis based on participants' choice switches is also reported below. Overall, these model-based results were in agreement with their model-free analogues such as choice switch probability following a win or a no-win outcome during RL (further details below).

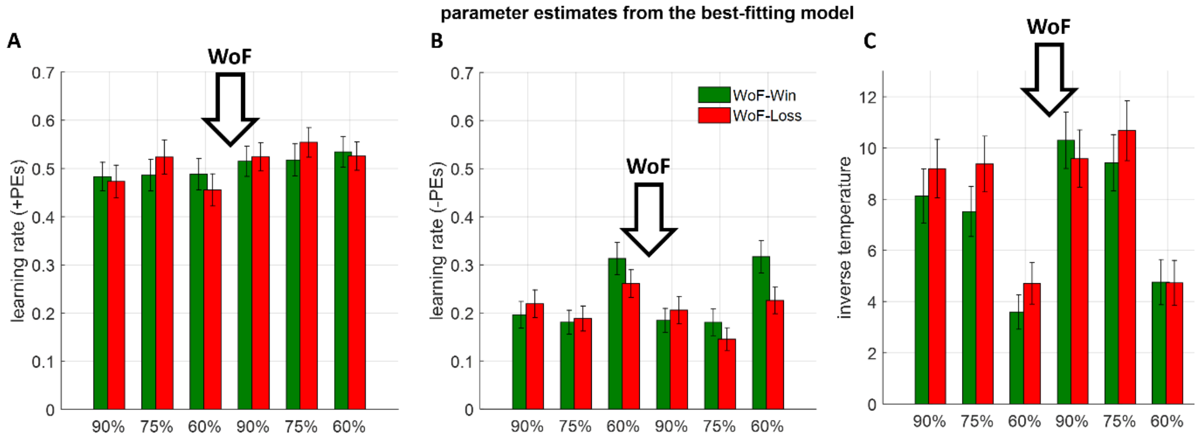

Figure S5. **Summaries of parameter estimates from the best-fitting reinforcement-learning model, related to Figure 2. A.)** Positive learning rates, by which participants update their beliefs about the reward probability following a reward outcome **B.)** Negative learning rates updating beliefs about the reward probability following a null/unrewarded outcome. **C.)** Inverse temperature term which governs choice stochasticity during reinforcement learning. Subsequent statistical analysis indicate that discrete affective events more strongly influence negative learning rates and choice stochasticity. Downward arrows with WoF indicate the point in which participants experienced the WoF draw within the course of their daily learning sessions. Therefore, across all panels, bars on the left side of the downward WoF arrows refer to pre-WoF blocks, whereas the bars on the right side of the arrows refer to the post-WoF blocks. Across all panels, error bars reflect ±1 SEM. Results from a complementary model-free analysis focusing on participants probability of choice switches are reported as shown in Supplementary Figure 5.'

Model-free control analysis of WoF effects on reinforcement learning

We conducted a model-free control analysis on the effects of WoF outcome on human learning behaviour, focusing on participants' probability of choice switch following a "win" or a "no-win" outcome across different probability levels and pre-and post-WoF. In this analysis, probability of switching choice after a win outcome would be similar to the positive learning rates, whereas probability of switching choice after a no-win outcome would be similar to negative learning rates. Here is worthwhile to highlight that, this model-free analysis approach cannot capture the choice stochasticity effects, which are captured by the best fitting RL model. As a result, inherent choice stochasticity commonly observed in human behaviour can confound the model-free analysis of choice switches to a certain degree.

We analysed participant choice behaviour by fitting a comparable 2x3x2 rmANOVA model to choice switch probabilities following a win outcome, which indicated a significant main effect of WoF phase ($F(1,65) = 6.018$, $p = .017$) a significant main effect of WoF outcome valence ($F(1,65) = 4.175$, $p = .045$). There was no significant interaction between these factors ($F(1,65) = 2.894$, $p = .094$) and no main effect of WoF order ($F(1,65) = .871$, $p = .354$). There was a

significant main effect of winning in the WoF on choice switch probabilities, which were consistently lower in the post-WoF blocks (F(1,65) = 7.488, p =.008, **Figure S6A**).

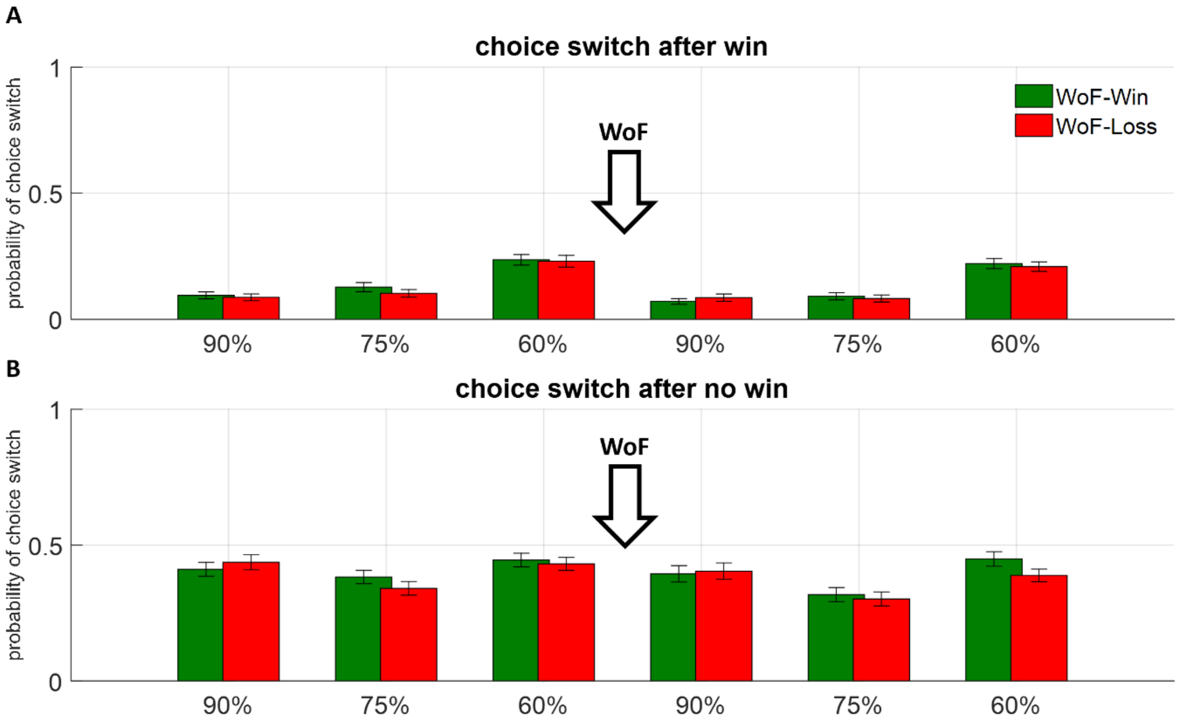

Figure S6. **Model-free analysis of participant learning behaviour, related to Figure 2. A.)** Probability of choice which following a win outcome. **B.)** Probability of choice which following a no-win outcome. Downward arrows with WoF indicate the point in which participants experienced the WoF draw within the course of their daily learning sessions. Across both panels, error bars reflect ±1 SEM.

Analysing the probability of choice switch after a no-win outcome in the same manner indicated a significant main effect of WoF phase (F(1,65) = 12.649, p <.001). There was also no significant main effect of WoF outcome valence (F(1,65) = 2.288, p = .135). There was no significant interaction between these factors (F(1,65) = .613, p = .436) and no main effect of WoF order (F(1,65) = .661, p = .419). In line with the model-based analysis indicating lower negative learning rates after losing in the WoF draw, there was a significant main effect of losing in the WoF on choice switch probabilities, which were consistently lower in the post-WoF blocks (F(1,65) = 10.756, p =.002, **Figure S6B**).

Model-free control analysis of WoF effects on preference tests

Human value-based recall is stable between 24 and 48h assessments.

In Study 1, we administered the preference test on 2 consecutive days. We analysed the frequency of shapes chosen in a 2x3x2 rmANOVA (i.e. 2 preference days, 3 WoF outcome valence, 2 probability levels [high versus low]) while controlling for WoF outcome order. There was no main effect of test day on participant choice behaviour (i.e. preference day 1 versus day 2, F(1,39 = .447, p = .508), indicating that value-based decision-making between affective memories remained stable between 24 and 48h after the experience of the last affective event (i.e. day 3 WoF outcome). Computed test-retest reliability coefficient for this measure was 0.883.

WoF effects feed forward to influence recall for post-WoF shapes.

In Study 2 in which we had a fully balanced design (Table 2) in terms of the probabilities of RL blocks before and after the WoF draw, we were able confirm that the effect of WoF feeds forward more stringently. We fitted a 2x2x2 (valence x phase [i.e. pre-versus post-WoF shapes] x reward probability) rmANOVA model. This analysis reconfirmed the main effect of reward probability (F (1, 69) = 66.623, p<.001, in line with results in the main text). There was no main effect of WoF outcome valence (F (1, 69) = 0.421, p = .518). However, there was a significant main effect of WoF phase (F (1, 69) = 6.329, p= .014) and only a marginally significant interaction between WoF phase and reward probability (F (1, 69) = 3.608, p =.062). There was no 3-way interaction between reward probability, WoF phase and WoF outcome valence (F (1, 64) = 0.013, p = .910). Irrespective of the WoF outcome valence, post-WoF shapes were preferred over pre-WoF shapes (t(146) = 3.352, p = .001), confirming that the affective influence of the WoF outcome feeds forward.

Supplementary Tables and Figures

Table S1. Equal value comparisons in the lab-based study, related to Figure 3

	Day 1		Day 2	
**Comparisons**	**t-statistic**	***p*-value**	**t-statistic**	***p*-value**
Win 90% vs Loss 90%	0.36	.72	.51	.61
Win 10% vs Loss 10%	-.48	.64	-.31	.76
Win 80% vs Loss 80%	.62	.54	.57	.57
Win 20% vs Loss 20%	-2.95	.005	-5.45	<.001*
Loss 90% vs Neutral 90%	2.94	.005	2.37	.02
Loss 10% vs Neutral 10%	-2.57	.01	-2.34	.02
Loss 80% vs Neutral 80%	.87	.39	0.22	.83
Loss 20% vs Neutral 20%	1.80	.08	2.49	.017
Win 90% vs. Neutral 90%	4.79	< .001*	4.19	<.001*
Win 10% vs. Neutral 10%	-2.52	.02	-2.21	.03
Win 80% vs. Neutral 80%	1.65	.11	.98	.34
Win 20% vs. Neutral 20%	-2.13	.04	-2.89	.006

df = 43, t-test pairwise comparisons, *.0042 (set level for 12 multiple comparisons Bonferroni corrected). Win 90% versus Neutral 90% is the only pairwise comparison which would survive Bonferroni correction over 2 preference tests.

Table S2. First, 2nd and 3rd quartile values of model parameters, related to Figure 3

Studies	win_gamma	win_delta	loss_gamma	loss_delta
**Study 1, Day 1 (25%; median;75%)**	1.008; 2.096;2.526	.908;2.318;3.181	.523;1.448;2.410	.321;.739;2.442
**Study 1, Day 2 (25%; median;75%)**	1.438;2.327;2.497	1.286;2.041;3.286	.553;1.486;2.390	.308;.759;2.247
**Study 2 (25%; median;75%)**	.933;2.588;3.038	1.509;2.262;3.384	1.175;2.381;3.085	1.031;2.189;2.633

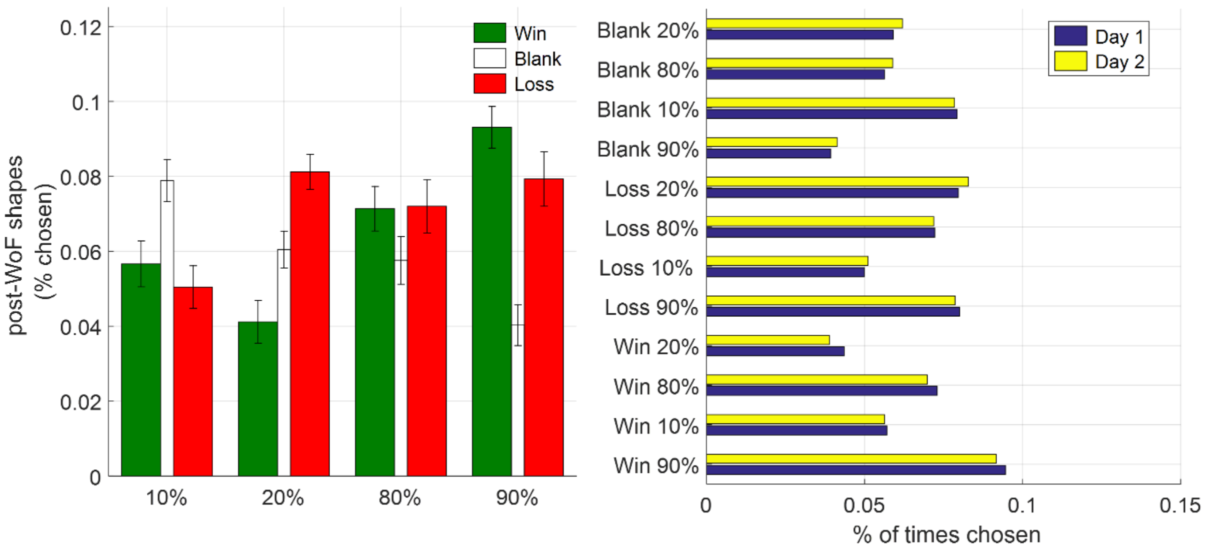

Figure S7. Chosen shape types in the lab-based study, related to Figure 3. **A.)** Overall summary of chosen post-WoF shapes averaged across day 1 and day 2, sorted by WoF outcome valence and reward probability. Under each WoF outcome valence category (i.e. win [green], loss [red], blank non-affective shapes [thinner white bars]). Analysis of this data with an rmANOVA model indicated a significant main effect of reward probability and a significant reward probability by WoF outcome valence interaction also reported in the main body of the article. Follow-up analysis indicated a main effect of valence (within high probability shapes: $F(2, 78) = 10.475$, $p<.001$; within low probability shapes $F(2,78) = 3.690$, $p=.029$). Error bars denote ±1 SEM. Qualitatively, the crossover between win and loss post-WoF shapes from low to high probability range overlap with the probability weighting curves reported in Figure 2. Note that these model-free analyses cannot take inherent choice stochasticity into consideration. **B.)** Detailed summary of how frequently individual shapes were chosen on each preference day. The increase in preference for low probability loss shapes reported in panel A appears to be driven by a stronger preference for Loss 20% shapes relative to Win 20% shapes. Pairwise comparisons between equal value shape pairs are summarised in Supplementary Table 1 and further analysis on the stability of choice preference between days 1 and 2 are reported in Supplementary Results in a dedicated section.

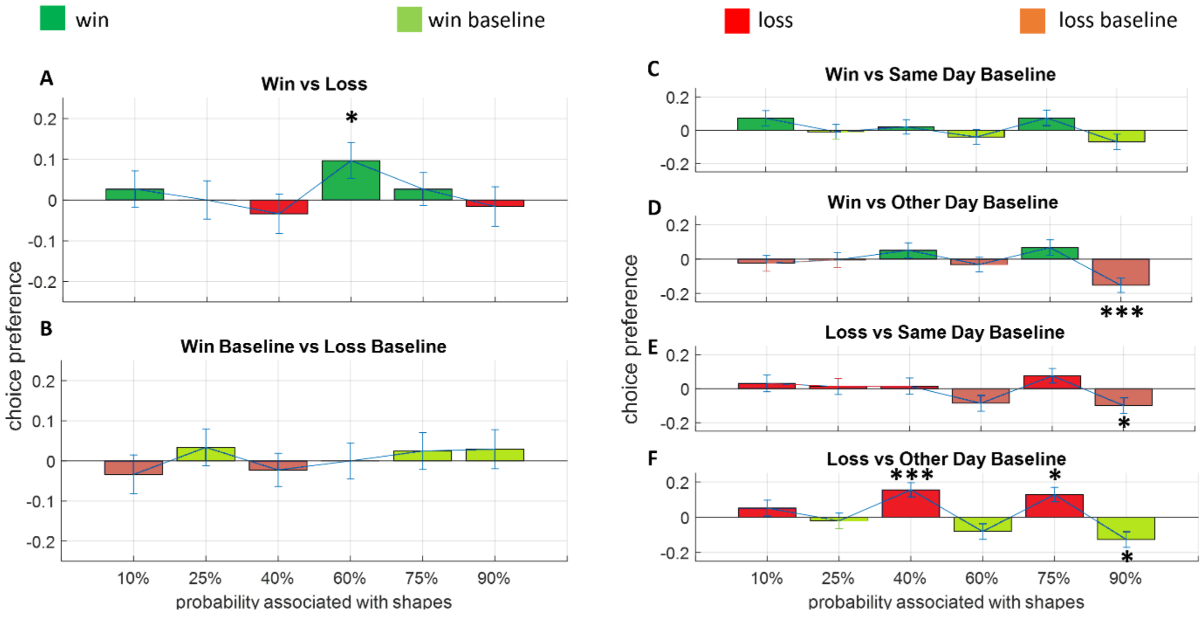

Figure S8. Comparisons between equal value shapes in the online study, related to Figure 3. A.) Shapes learned after a win or a loss WoF draw**. B.)** Shapes learned prior to a win or a loss WoF draw. **C-F.)** Shapes learned before versus after a win or a loss WoF draw including same and other day baseline comparisons. Values across all panels are normalised, meaning that positive values on the y-axis indicate a preference for affective (i.e. post-WoF) shapes. Across all panels, error bars reflect ±1 SEM. ***p<.001, *p<.05, uncorrected. All reward probabilities are reordered in linearly increasing order. Bar graphic face colours follow the legend above top panels.

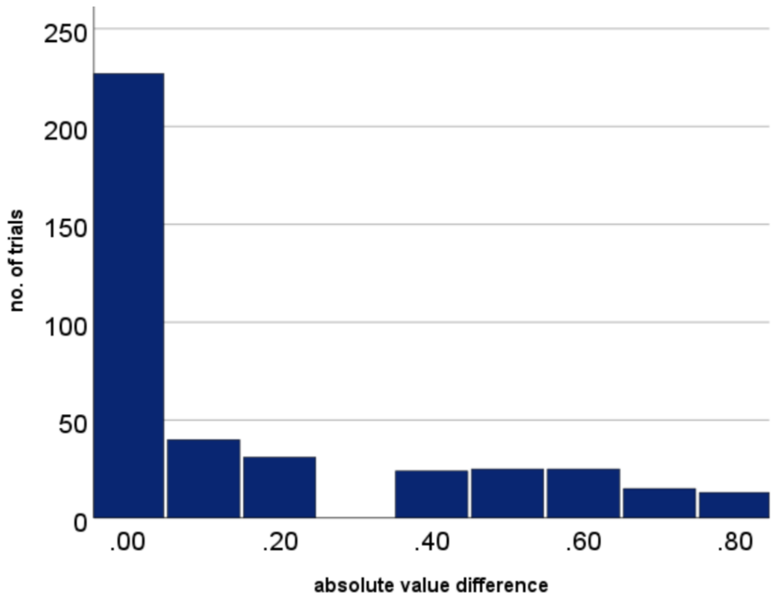

Figure S9. **Absolute value difference between shape options presented in the preference test, related to STAR Methods.** Trials in the preference test were allocated to maximise the number of equal reward probability outcomes (i.e. where the absolute value difference between presented shapes is zero). This is computed irrespective of WoF outcome valence.

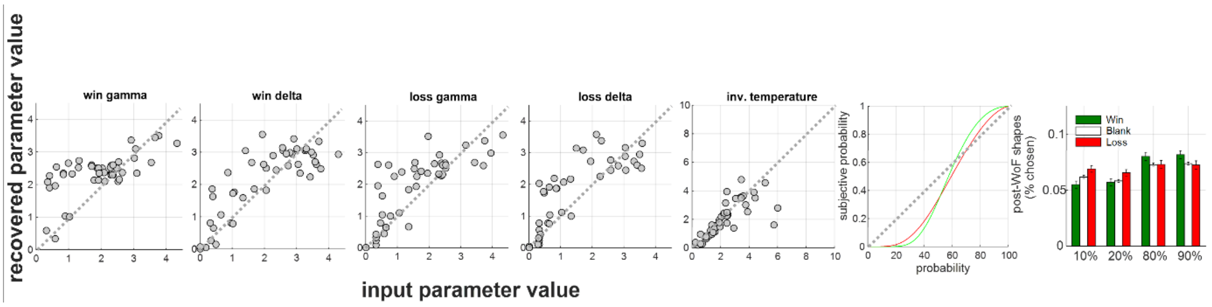

Figure S10. **Parameter recovery results for the memory-guided value-based decision-making model, related to Figure 3.** Using the values observed in our cohort (e.g. values plotted in Figure 3A) and a randomly selected participant's preference test trial sequence as inputs, we performed parameter simulations with stochastic choice (i.e. in the same manner as models were fitted, e.g. using MATLAB's *randsample* function choosing option 1=randsample([1 0],1,1,[option_1_prob (1-option_1_prob)]). All recovered parameters were significantly correlated with actual input parameters (.62<r<.8315, all p<.001, first 5 panels from left). The summary of the recovered parameters continues to capture the crossover in the probability weighting function from low to high probability range (6th panel from right), and choices generated stochastically from the recovered parameters demonstrate the same probability x valence interaction effect from raw choices (post WoF-loss preference higher than post WoF-win preference in the low probability range, post WoF-win preference higher than post WoF-loss preference in the high probability range; rightmost panel).

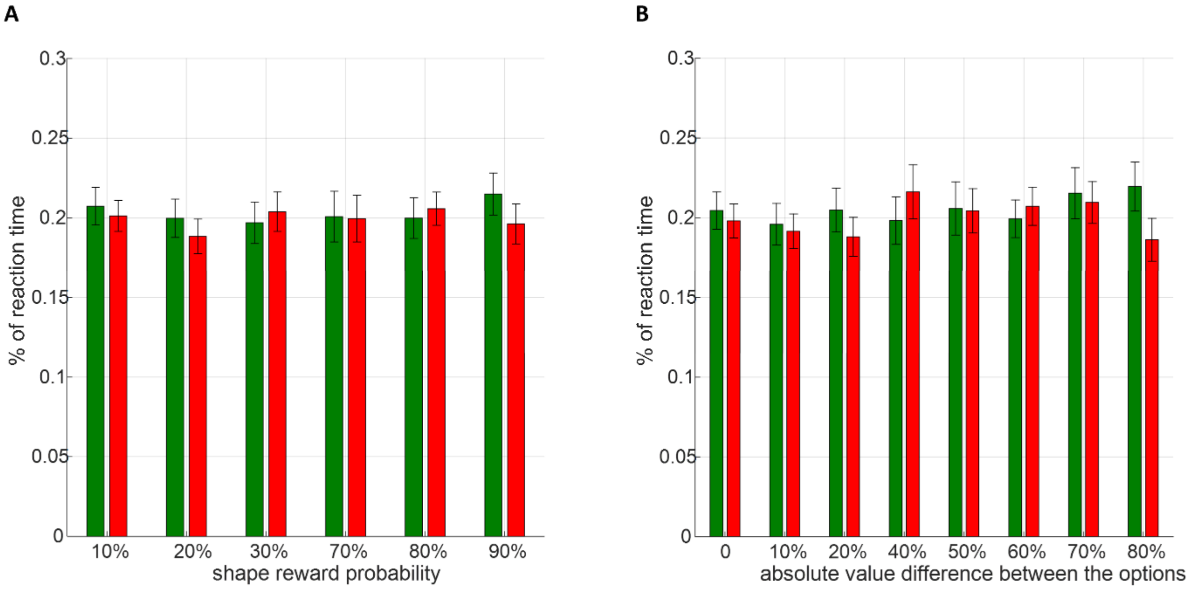

Figure S11. **Shape evaluation duration as a proportion of reaction time, irrespective of choice and unchosen options, related to Figure 4. A.)** Proportion of reaction time spent to evaluate the shapes prior to decision-onset, bars plotted as a function of shape reward probability. **B.)** The same analysis with respect to absolute value difference between the shapes. Pre-WoF baseline shapes (i.e. 70/30%) included to minimise number of trials excluded.

Supplementary References

1. Rescorla, R.A., and Wagner, A.R. (1972). A theory of Pavlovian conditioning: Variations in the effectiveness of reinforcement and nonreinforcement. Classical conditioning II: Current research and theory *2*, 64-99.
2. Michely, J., Eldar, E., Martin, I.M., and Dolan, R.J. (2020). A mechanistic account of serotonin's impact on mood. Nature communications *11*, 1-11.
3. Rouhani, N., Norman, K.A., Niv, Y., and Bornstein, A.M. (2020). Reward prediction errors create event boundaries in memory. Cognition *203*, 104269.
4. Burnham, K.P., and Anderson, D.R. (2003). Model selection and multimodel inference: a practical information-theoretic approach (Springer Science & Business Media).
5. Prelec, D. (1998). The probability weighting function. Econometrica, 497-527.
6. Daw, N.D. (2011). Trial-by-trial data analysis using computational models. Decision making, affect, and learning: Attention and performance XXIII *23*, 1.
7. Pulcu, E., and Browning, M. (2017). Affective bias as a rational response to the statistics of rewards and punishments. Elife *6*.
8. Pulcu, E., and Haruno, M. (2019). Value computations underlying human proposer behavior in the ultimatum game. Journal of Experimental Psychology: General.
